# The Abl-interactor Abi suppresses the function of the BRAG2 GEF family member Schizo

**DOI:** 10.1242/bio.058666

**Published:** 2024-01-05

**Authors:** Stefanie Lübke, Carina Braukmann, Karl-Heinz Rexer, Lubjinka Cigoja, Pratiti Rout, Susanne F. Önel

**Affiliations:** ^1^ Fachbereich Medizin, Department for Molecular Cell Physiology, Institute for Physiology and Pathophysiology, Philipps-Universität Marburg, Emil-Mannkopff-Str. 2, 35037 Marburg, Germany; ^2^ Fachbereich Biologie, Department for Developmental Biology, Philipps-Universität Marburg, Karl-von-Frisch-Str. 8, 35043 Marburg, Germany; ^3^ DFG Research Training Group, Membrane Plasticity in Tissue Development and Remodeling, GRK 2213, Philipps-Universität Marburg, Marburg, Germany; ^4^ Fachbereich Biologie, Department for Biodiversity of Plants, Philipps-Universität Marburg, Karl-von-Frisch-Str. 8, 35043 Marburg, Germany; ^5^ Fachbereich Biologie, Department for Molecular Embryology, Philipps-Universität Marburg, Karl-von-Frisch-Str. 8, 35043 Marburg, Germany

**Keywords:** Axon guidance, Graf-1, Myoblast fusion, N-Cadherin, Slit, Sec7

## Abstract

Guanine nucleotide exchange factors (GEF) of the BRAG subfamily activate small Arf GTPases, which are pivotal regulators of intracellular membrane traffic and actin dynamics. Consequently, BRAG proteins have been implicated to regulate the surface levels of adhesive and signaling receptors. However, not much is known about the mechanism leading to the regulation of these surface proteins. In this study, we found that the *Drosophila* BRAG GEF Schizo interacts physically with the Abl-interactor (Abi). *schizo* mutants display severe defects in myoblast fusion during syncytial muscle formation and show increased amounts of the cell adhesion protein N-cadherin. We demonstrate that the *schizo* myoblast fusion phenotype can be rescued by the expression of the Schizo GEF (Sec7) and membrane-binding (pleckstrin homology) domain. Furthermore, the expression of the Sec7-PH domain in a wild-type background decreases the amounts of N-cadherin and impairs myoblast fusion. These findings support the notion that the Sec7-PH domain serves as a constitutive-active form of Schizo. Using a yeast-two hybrid assay, we show that the SH3 domain of Abi interacts with the N-terminal region of Schizo. This region is also able to bind to the cytodomain of the cell adhesion molecule N-cadherin. To shed light on the function of Schizo and Abi in N-cadherin removal, we employed epistasis experiments in different developmental contexts of *Drosophila*. These studies point towards a new model for the regulation of Schizo. We propose that the binding of Abi to the N-terminal part of Schizo antagonizes Schizo function to inhibit N-cadherin removal.

## Teaser

**Summary:** The Drosophila GEF Schizo removes N-cadherin during myoblast fusion. We found that Schizo interacts with Abi to antagonize Schizo function and to inhibit the removal of N-cadherin during embryogenesis.

## INTRODUCTION

BRAG proteins are a subgroup of the Arf-guanine nucleotide exchange factor (GEF) family that are mandatory for developmental and physiological processes, e.g. myoblast fusion, neuronal pathfinding and synaptic transmission. However, they also play an important role during disease progression, e.g. cancer metastasis and X-chromosome-linked intellectual disability (D'Souza and Casanova, 2016). The BRAG family is characterized by an N-terminal located calmodulin-binding IQ motif, a catalytic Sec7 domain of ∼200 amino acids and a pleckstrin homology (PH) domain that is immediately located downstream of the Sec7 domain. The Sec7 domain stimulates the release of GDP to allow binding of GTP on ADP-ribosylation factor (Arf) family members. These Arf GTPases serve as master regulators of intracellular membrane traffic and actin dynamics. A primary challenge in understanding the activation of small GTPases in development, tissue homeostasis and disease is to study the molecular mechanism underlying GEF activation.

The human GEFs for Arf family proteins are grouped into six evolutionarily conserved families known as BIG, BRAG/IQSec, Cytohesins, EFA6/PSD, FBX8 and GBFs. Arf GEFs of the Cytohesin and BIG family are regulated by auto-inhibition (DiNitto et al., 2007; Richardson et al., 2012; Stalder et al., 2011). The GEF activity of Cytohesin is suppressed through the Sec7-PH linker and the C-terminal helix/polybasic region that mask the active side in the Sec7 domain (DiNitto et al., 2007). The auto-inhibition of the yeast BIG family member Sec7 depends on an intramolecular interaction that involves the HSD domains (Richardson et al., 2012). Auto-inhibition is released by the binding of membrane-bound Arf-GTP to the PH domain or the HSD1 domain. A positive feedback loop arises through the generation of more Arf-GTP by the Sec7 GEF, which leads to the recruitment of more Arf-GEF. In contrast, high resolution crystal structure of the unbound Sec7-PH domain of the Sec7 GEF BRAG2 revealed that the lipid-binding side in the PH domain and the Arf-binding site in the Sec7 domain are both constitutively active (Karandur et al., 2017). The finding that the Sec7 and PH domains are constantly accessible for protein­–protein and protein–membrane interactions raises the question of how the spatial–temporal activation of BRAG2 is achieved.

The *Drosophila* BRAG family member Schizo is required for the guidance of neuronal axons (Önel et al., 2014) and muscle development (Chen et al., 2003; Dottermusch-Heidel et al., 2012). Muscles are multinucleated cells that arise by the fusion of mononucleated myoblasts. Besides muscle formation, myoblast fusion is crucial for the maintenance, growth and repair of muscles in mammals and *Drosophila* (Abmayr and Pavlath, 2012; Chaturvedi et al., 2017). However, the precise function of Schizo during myoblast fusion is still unknown. Rescue experiments with *Drosophila* Arf1, Arf2 and Arf6 that represent the three classes of mammalian Arf GTPases (Donaldson and Jackson, 2011), suggest that Schizo acts through the Arf1-GTPase (Dottermusch-Heidel et al., 2012). In a global yeast two-hybrid screen the cell adhesion molecule N-cadherin was identified as Schizo interaction partner (Dottermusch-Heidel et al., 2012). Genetic interaction studies revealed that the *schizo* myoblast fusion phenotype is suppressed by the loss of N-cadherin. Based on these findings we proposed that the removal of N-cadherin brings the apposing myoblast membranes into close proximity to allow membranes to fuse.

In this study, we have analyzed the function of the N-terminal domain of Schizo. We found that the expression of Schizo lacking this N-terminal domain in a wild-type background leads to a nuclear localization of Schizo. However, rescue experiments suggest that this truncation is still able to rescue the *schizo* myoblast fusion phenotype. Furthermore, we demonstrate that the *schizo* mutant phenotype can be rescued by the expression of the Sec7-PH domain during muscle development. These findings prove that the Sec7-PH domain serves as a constitutive-active form of Schizo during development. Accordingly, the expression of the Sec7-PH domain in a wild-type background reduces amounts of N-cadherin. To understand by which mechanism amounts of N-cadherin are regulated, we have investigated whether Graf-1 dependent CLIC/GEEC endocytosis is involved in this process. However, neither a *graf-1* mutant generated by CRISPR/Cas9 nor the expression of truncated Graf-1 showed *schizo*-like phenotypes or increased amounts of N-cadherin. Instead, we found that the Scar/WAVE complex member Abi binds to the N-terminal region of Schizo. Abi, but not Scar/WAVE, antagonizes Schizo function in a dosage-dependent manner. These findings provide a new conceptual framework for the regulation of Schizo activity.

## RESULTS

### The N-terminal region of Schizo is important for protein localization

**Fig. 1.**
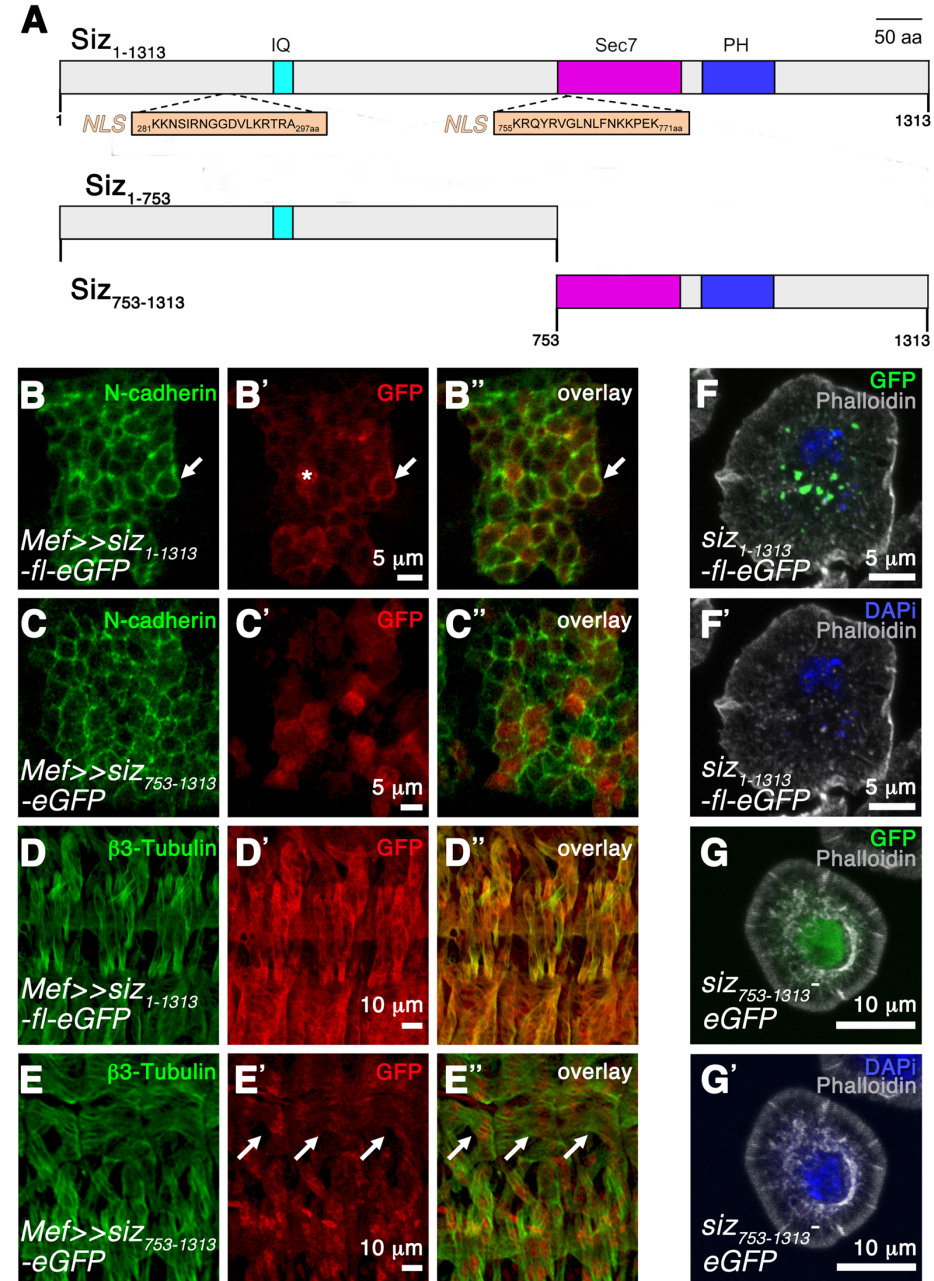
**Schizo is present at the plasma membrane together with N-cadherin in the presence of the N-terminal region, but localizes to the nucleus in the absence of this region.** (A) Schematic representation of the domain structure of Schizo P2 full-length Siz_1-1313_, Siz_1-753_ containing only the N-terminal region and Siz_753-1313_ lacking the N-terminal region. Schizo contains three evolutionarily conserved domains: a Calmodulin domain (IQ), a Sec7 domain and a pleckstrin homology domain (PH). Two nuclear localization sequences (NLS) are predicted within the Schizo sequence and are located before the IQ and in the Sec7 domain. (B–B″). Higher magnification of a stage 10 embryo expressing Siz_1-1313_-eGFP with Mef-GAL4 in the mesoderm. The embryo was stained with anti-GFP to follow Siz_1-1313_-eGFP expression and with anti-N-cadherin, which is present at the plasma membrane. Siz_1-1313_-eGFP is detectable in the cytoplasm (asterisks) and around the myoblast (arrow). Scale bars: 5 µm. (C–C″) Higher magnification of a stage 10 embryo expressing Siz_753-1313_-eGFP with Mef2-GAL4 in the mesoderm. The embryo was stained with anti-GFP and anti-N-cadherin. In the absence of the N-terminal region, Siz_753-1313_-eGFP is only present in the cytoplasm and not at the plasma membrane as previously observed in embryos expressing Siz_1-1313_-eGFP. Scale bars: 5 µm. (D–D″) Higher magnification of a stage 16 embryo expressing Siz_1-1313_-eGFP with Mef2-GAL4 in muscles. The embryo was stained with anti-GFP to follow Siz_1-1313_-eGFP expression and with anti-β3-Tubulin to highlight somatic muscles. Most of the Siz_1-1313_ protein is present in the cytoplasm of muscle cells and at the plasma membrane. Scale bar: 10 µm. (E–E″) Higher magnification of a stage 16 embryo expressing Siz_753-1313_-eGFP. The embryo was stained with anti-GFP and anti-β3-Tubulin. The cytoplasmic distribution of Siz_753-1313_ in muscle cells seems to be reduced in comparison to Siz_1-1313_. Instead, distinct spots are visible (arrows). (F–F′) Transfection of *Drosophila* S2R+ cells with Siz_1-1313_-eGFP. Cells were plated on concanavalin A coated cover slips and stained for DAPi (blue) and Phalloidin (grey). Siz_1-1313_ is distributed in a punctuated manner. Scale bars: 5 µm. (G–G′) Transfection of *Drosophila* S2R+ cells with Siz_753-1313_-eGFP. Cells were plated on concanavalin A-coated cover slips and stained for DAPi (blue) and Phalloidin (grey). In the absence of the N-terminal region Siz_753-1313_ is detectable in the nucleus. Scale bars: 10 µm.

*schizo* encodes for two Arf GEFs (Schizo P1 and Schizo P2) that only differ in the first 12 amino acids at their N-terminal region and share all the conserved domains (Önel et al., 2004). These Schizo proteins correspond to the Loner isoforms Iso1 and Iso2 that have been described by Chen et al. (2003). In Dottermusch-Heidel et al. (2012) we screened a *Drosophila* yeast two-hybrid cDNA library with the first 753 amino acids of Schizo P2 (Fig. 1A) and identified N-cadherin as interaction partner. To determine whether the N-terminal region of Schizo is important for Schizo P2 localization, we placed the wild-type *schizo* cDNA LP01489 lacking the first 2,3 kb of the *schizo* ORF under the control of UAS activating sequences. The full-length Schizo protein P2 Siz_1-1313_, the Schizo protein Siz_1-753_ from the yeast-two hybrid screen and Siz_753-1313_ lacking the first 2,3 kb of the LP01489 *schizo* cDNA are shown in Fig. 1A. GFP-tagged Siz_1-1313_ and Siz_753-1313_ were expressed in the mesoderm and in somatic muscle cells with the *Mef2-*GAL4 driver. In the mesoderm of stage 10 embryos, Siz_1-1313_ and N-cadherin are both present at the plasma membrane (Fig. 1B–B″ arrow), but Siz_1-1313_ is also detectable in the cytoplasm (Fig. 1B–B″ asterisks and D–D″). Since the N-cadherin antibody detects the extracellular region of N-cadherin, Siz_1-1313_ and Ncadherin do not co-localize in Fig. 1B–B″. In contrast, Siz_753-1313_ lacking the N-terminal region is only detectable in the cytoplasm and not at the plasma membrane like observed for Siz_1-1313_ (Fig. 1C–C″). In somatic muscles Siz_753-1313_ is expressed in a very specific pattern that is reminiscent of the nuclear expression pattern of the transcription factor Mef2 (Fig. 1E′ and E″ arrows).

To confirm the different localization of Siz_1-1313_ full-length and truncated Siz_753-1313_, both proteins were expressed in *Drosophila* S2R+ Schneider cells (Fig. 1F,F′,G,G′ and Movie 1). Siz_1-1313_ is distributed in the cytoplasm of S2R+ cells in a punctuated manner (Fig. 1F and F′). The act that Siz_1-1313_ is not found at the plasma membrane is probably due to the overexpression situation. As suspected from the localization in Fig. 1E′ and E″, we observed a co-localization of Siz_753-1313_ with DAPi in *Drosophila* S2R+ cells confirming the nuclear localization of Siz_753-1313_ (Fig. 1G and G′). The import of proteins into the nucleus depends on a short peptide sequences called nuclear localization signal (NLS) sequences. To analyze whether Schizo P2 contains such NLS sequences, we have performed a data-bank search as described by Lin and Hu (2013) and identified two predicted NLS sequences in Siz_1-1313_ (Fig. 1A). One of these predicted NLS sequences is located in the Sec7 domain of Schizo P2 and is still present in Siz_753-1313_. However, the expression of Siz_753-1313_ in the mesoderm of wild-type embryos and its nucleocytoplasmic shuttling does not disturb myoblast fusion. Taken together, these results show that the N-terminal region of Schizo P2 is essential for the localization of the Schizo protein to the plasma membrane. In the absence of this region Schizo translocates to the nucleus although the Sec7 and PH domain that mediate lipid binding are still present.

### N-cadherin amounts are increased in homozygous *schizo* mutant embryos

**Fig. 2.**
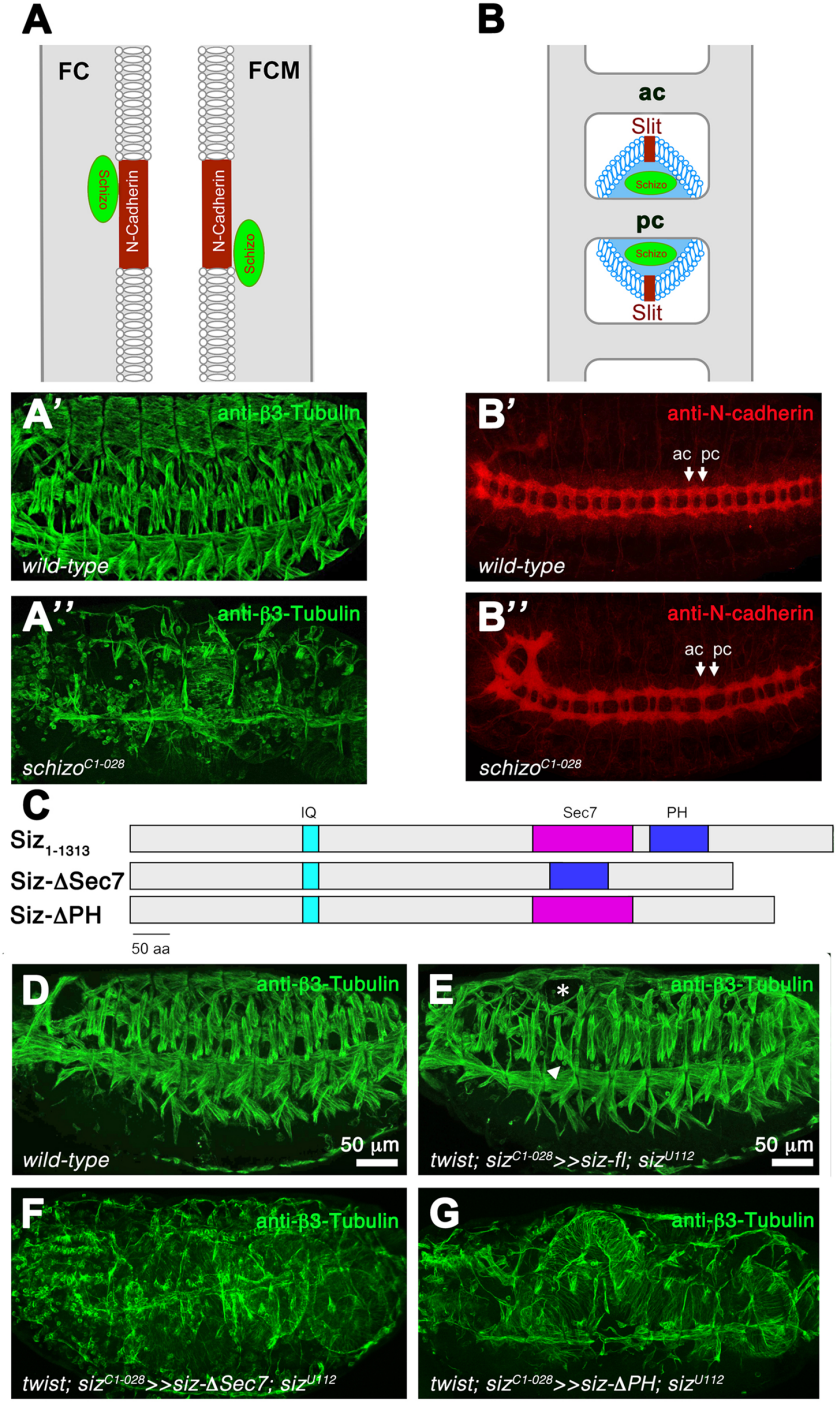
**The Sec7 and PH domains are important for Schizo function.** (A) Schizo is expressed in founder cells (FC) and fusion-competent myoblasts (FCM) and interacts with the cell adhesion protein N-cadherin. (A′ and A″) Lateral view of stage 16 embryos stained with anti-β3-Tubulin to visualize somatic muscles. (A′) In wild-type embryos, a repeated pattern of multinucleated muscles per hemisegment is visible at the end of embryogenesis. (A″) Homozygous *schizo^C1-028^* embryo showing unfused myoblasts. (B) During the development of the central nervous system, Schizo is exressed in midline glia cells and controls commissure formation by regulating Slit. (B′ and B″) Ventral view of stage 16 embryos stained with anti-N-cadherin to label all central axons. Anterior is left. Ac, anterior commissure, pc, posterior commissure. (B′) Wild-type. (B″) Missing posterior commissures are observed in homozygous *schizo^C1-028^* mutant embryos. (C) Schematic diagrams of the domain organization in Siz_1-1313_, SizΔSec7 and SizΔPH. Lateral view of stage 16 wild-type control embryo stained with anti- β3-Tubulin. (D–G) Lateral view of stage 16 embryos stained with anti-β3-Tubulin. (D) Wild type. (E) Transheterozygous *schizo^C1-028^/schizo^U112^* mutant embryo expressing *schizo* full-length Siz_1-1313_ in the mesoderm with *twist*-GAL4. (F) Transheterozygous *schizo^C1-028^/schizo^U112^* mutant embryo expressing SizΔSec7 fails to restore myoblast fusion defects. (G) Expression of SizΔPH in transheterozygous *schizo^C1-028^/schizo^U112^* mutant embryo fails to rescue the *schizo* mutant phenotype.

Schizo function is required in the two types of *Drosophila* myoblasts: founder cells (FCs) and fusion-competent myoblasts (FCMs) (Dottermusch-Heidel et al., 2012), where it interacts with N-cadherin. The loss of Schizo function leads to severe defects in myoblast fusion (Fig. 2A-A″). In the CNS, *schizo* mutants lack commissural axons (Fig. 2B-B″, arrows) due to increased levels of the axon guidance molecule Slit (Önel et al., 2004). To assess the importance of the Sec7 and PH domain for Schizo P2 function, we generated Siz-ΔSec7 and Siz-ΔPH deletion mutants (Fig. 2C). Expression of Siz_1-1313_ in the mesoderm with *twist*-GAL4 rescues the myoblast fusion phenotype (Fig. 2D and E), whereas homozygous mutants expressing Siz-ΔSec7 or Siz-ΔPH in their mesoderm still showed a myoblast fusion phenotype (Fig. 2F and G). These data confirm that the Sec7 and PH domains are both pivotal for Schizo function.

**Fig. 3.**
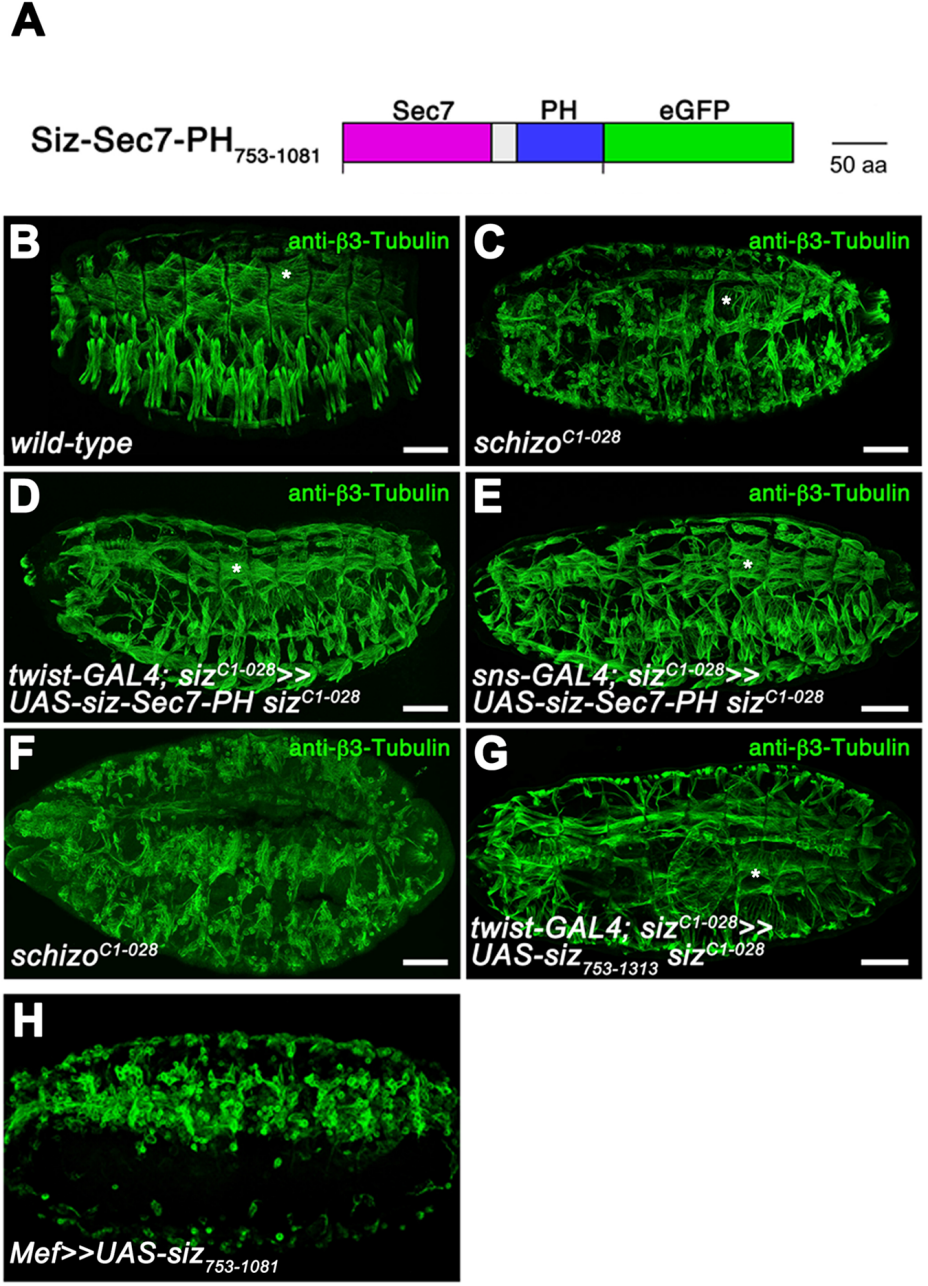
**Expression of the Sec7-PH module of Schizo and UAS-siz_752-1313_ in myoblasts rescues the *schizo* mutant phenotype.** (A) Schematic representation of Schizo containing only the Sec7-PH domain: Siz_753-1081_. (B–E) Dorsolateral view of stage 16 embryos stained with β3-Tubulin. (B) Wild-type embryo. (C) Homozygous *schizo^C1-028^* mutant embryo. (D) Homozygous *schizo^C1-028^* mutant embryo expressing the Sec7-PH module UAS-*siz_753-1081_* under the control of *twist*-GAL4. (E) Homozygous *schizo^C1-028^* mutant embryo expressing UAS-*siz_753-1081_* under the control of *sns*-GAL4. (F and G) Dorsal views of homozygous *schizo^C1-028^* mutant embryos showing that the expression of (G) UAS-siz_753-1313_ partially rescues the *schizo* mutant phenotype. Scale bar: 50 µm. (H) Anti-β3-tubulin staining. Lateral view of stage 16 embryo expressing UAS-siz_753-1081_ with Mef-GAL4 in a wild-type background.

Studies on the Schizo homologue BRAG2 have demonstrated that the Sec7-PH module possesses a 10-fold higher activity towards Arf1 and a 15–20-fold higher activity in the presence of PIP2 (Jian et al., 2012; Aizel et al., 2013). To investigate whether the Schizo Sec7-PH module serves as an activated form of Schizo, we amplified the coding region of the Sec7-PH module Siz_753-1081_ and cloned it under the control of the UAS activating sequences (Fig. 3A). We observed that the expression of Siz_753-1081_ in FCs and FCMs with *twist*-GAL4 as well as the exclusive expression of Siz_753-1081_ in FCMs with *sns*-GAL4 is able to rescue the *schizo* myoblast fusion phenotype (Fig. 3D and E). Furthermore, we noticed that the overexpression of Siz_753-1081_ with *Mef*-GAL4 in wild-type embryos also disturbs myoblast fusion (Fig. 3H). To determine whether the altered localization of Siz_753-1313_ affects Schizo function, we also performed a rescue experiment with Siz_753-1313_. We found that the expression of Siz_753-1313_ in both myoblast-types with *twist*-GAL4 partially rescues the *schizo* mutant phenotype (Fig. 3G). However, the comparison of the dorsal muscle group in wild-type (Fig. 3B, asterisks), *schizo^C1-028^* (Fig. 3C, asterisk) and rescued embryos with Siz_753-1081_ (Fig. 3D and E, asterisk) or Siz_753-1313_ (Fig. 3G, asterisk) let suggest that the rescue capacity of Siz_753-1313_ differs from the rescue capacity of Siz_753-1081_.

**Fig. 4.**
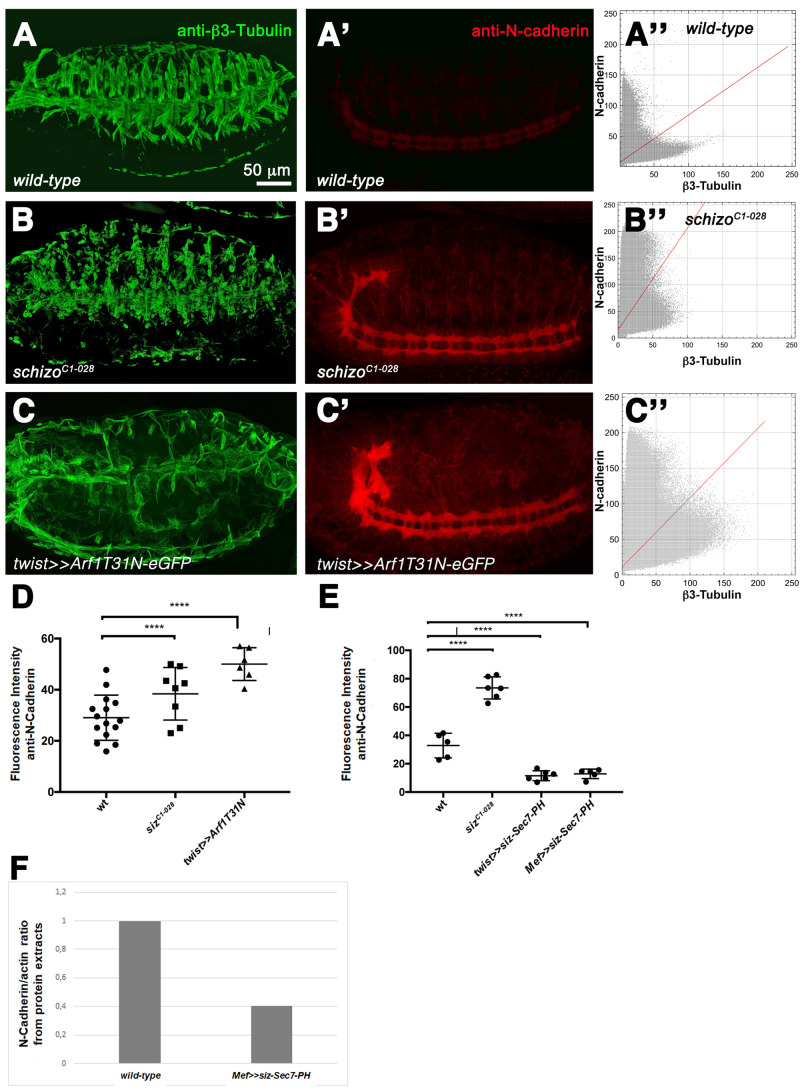
**Expression of the Schizo Sec7-PH domain decreases amounts of N-cadherin and induces myoblast fusion defects.** (A–C″ and C–C″). Lateral view of a stage 16 embryo stained with anti-β3-Tubulin (A–C and E) and anti-N-cadherin (A′–C′ and E′). (A″–C″ and E″) Cytoflourograms. (A,A′) Wild-type embryo. (A″) Cytofluorogram of the embryo imaged in A and A′ showing the fluorescence concentration of β3-Tubulin on the x-axis and N-cadherin on the y-axis. (B and B′) Homozygous *schizo^C1-028^* mutant embryo. (B″) Cytofluorogram of the embryo imaged in B and B′ showing the fluorescence concentration of β3-Tubulin on the x-axis and N-cadherin on the y-axis. Amounts of N-cadherin are increased. (C and C′) Expression of UAS-*Arf1T31N-eGFP* with *twist*-GAL4 in the background of a wild-type embryo. Myoblast fusion is disturbed in comparison to the wild type. (C″) Cytofluorogram of the embryo imaged in C and C′ showing the fluorescence concentration of β3-Tubulin on the x-axis and N-cadherin on the y-axis. Amounts of N-cadherin are increased. (D) Quantification of the amounts of N-cadherin in wild-type, homozygous *schizo^C1-028^* mutant embryos and embryos expressing UAS-*Arf1T31N-eGFP* with *twist*-GAL4. The total fluorescence intensity of 6 to 15 embryos was measured for each experiment. Bars represent mean±s.d. *P*-values were calculated using the Dunnett's multiple comparison test. p***< 0,0001 compared with wild type. (E) Quantification of the amounts of N-cadherin in wild-type, homozygous *schizo^C1-028^* mutant embryos, embryos expressing UAS-*siz-Sec7-PH-eGFP* with *Mef2-*GAL4 and *twist*-GAL4. The total fluorescence intensity of 5 to 6 embryos was measured for each experiment. In *Mef2*-GAL4>>UAS-*siz-Sec7-PH-eGFP* and *twist*-GAL4>>UAS-*siz-Sec7-PH-eGFP* embryos amounts of N-cadherin are decreased. Bars represent mean±s.d. *P*-values were calculated using the Dunnett's multiple comparison test. P***<0,0001 compared to wild type. (F) Verification of the data presented in E by determining the ratio of N-cadherin and actin on western blots using protein extracts from wild-type and Mef>>UAS-*siz_752-1081_*-expressing embryos.

Still the question remained whether the Sec7-PH module is crucial for regulating amounts of N-cadherin. To address this question, we first elevated the amount of N-cadherin in homozygous *schizo* mutants and in embryos expressing dominant-negative Arf1 (Arf1T31N) by using two different approaches. Consistent with our previous hypothesis that was based on genetic interaction studies, we observed increased amounts of N-cadherin in whole-mount embryos (Fig. 4A′,A″,B′,B″,C′ and C″). To analyze the amount of N-cadherin, we used cytofluorograms of single embryos (Fig. 4A″,B″ and C″) and measured the fluorescence intensity from 6 to 15 embryos (Fig. 4D). Additionally, we examined the N-cadherin fluorescence intensity of embryos expressing Sec7-PH_753-1081_ in the mesoderm with *twist*-GAL4 or with *Mef*-GAL4 in muscle cells. With both GAL4 drivers, we observed decreased amount of N-cadherin (Fig. 4E). Additionally, we validated decreased amounts of N-cadherin by Western blot analysis (Fig. 4F). Taken together, these data show that the Schizo full-length protein is required for the removal of N-cadherin and that Schizo-Sec7-PH_753-1081_ represents an activated form of Schizo.

### The Scar/WAVE complex member Abi interacts physically with the RhoGAP protein Graf-1 and Schizo

The finding that amounts of N-cadherin are decreased in Schizo-Sec7-PH_753-1081_ expressing embryos combined with our previous findings that N-cadherin is increased in the absence of *schizo*, prompted us to investigate whether Schizo controls N-cadherin amounts by endocytosis. The observation that the *schizo* mutant phenotype can be rescued by GTP-bound Arf1 suggests that Schizo acts via Arf1 (Dottermusch-Heidel et al., 2012). Consistently, we detect a transient colocalization between Siz_1-1313_ and Arf1 in *Drosophila* S2R+ cells (Fig. S1). The small Arf1-GTPase is involved in the endocytosis of lipid-anchored proteins such as GPI-APs and does not involve Dynamin. The endocytotic structures are termed GEECs (GPI-AP enriched early endosomal compartments). The molecular mechanism of this pathway is initiated by the recruitment of the Sec7 GEF GBF1, which activates Arf1 (Gupta et al., 2009). Arf1 recruits the RhoGAP protein Graf-2 (alias ARHGAP10, alias ARHGAP21) to the cell surface (Kumari and Mayor, 2008). The activity of this protein complex promotes GTP-hydrolysis on Cdc42, which is necessary for the endocytosis process. Additionally, Graf-1 (alias ARHGAP26) has been described to colocalize with activated Cdc42 and controls like Graf-2 Cdc42 activity (Lundmark et al., 2008). During muscle development, the downregulation of Graf-1 in murine C2C12 cells or the loss of Graf-1 or Graf-2 in primary myoblasts significantly reduces the capability of myoblasts to fuse (Doherty et al., 2011; Lenhart et al., 2014). Due to the role of Graf in GEEC endocytosis and its role in mammalian myoblast fusion, we examined Graf function in *Drosophila*.

**Fig. 5.**
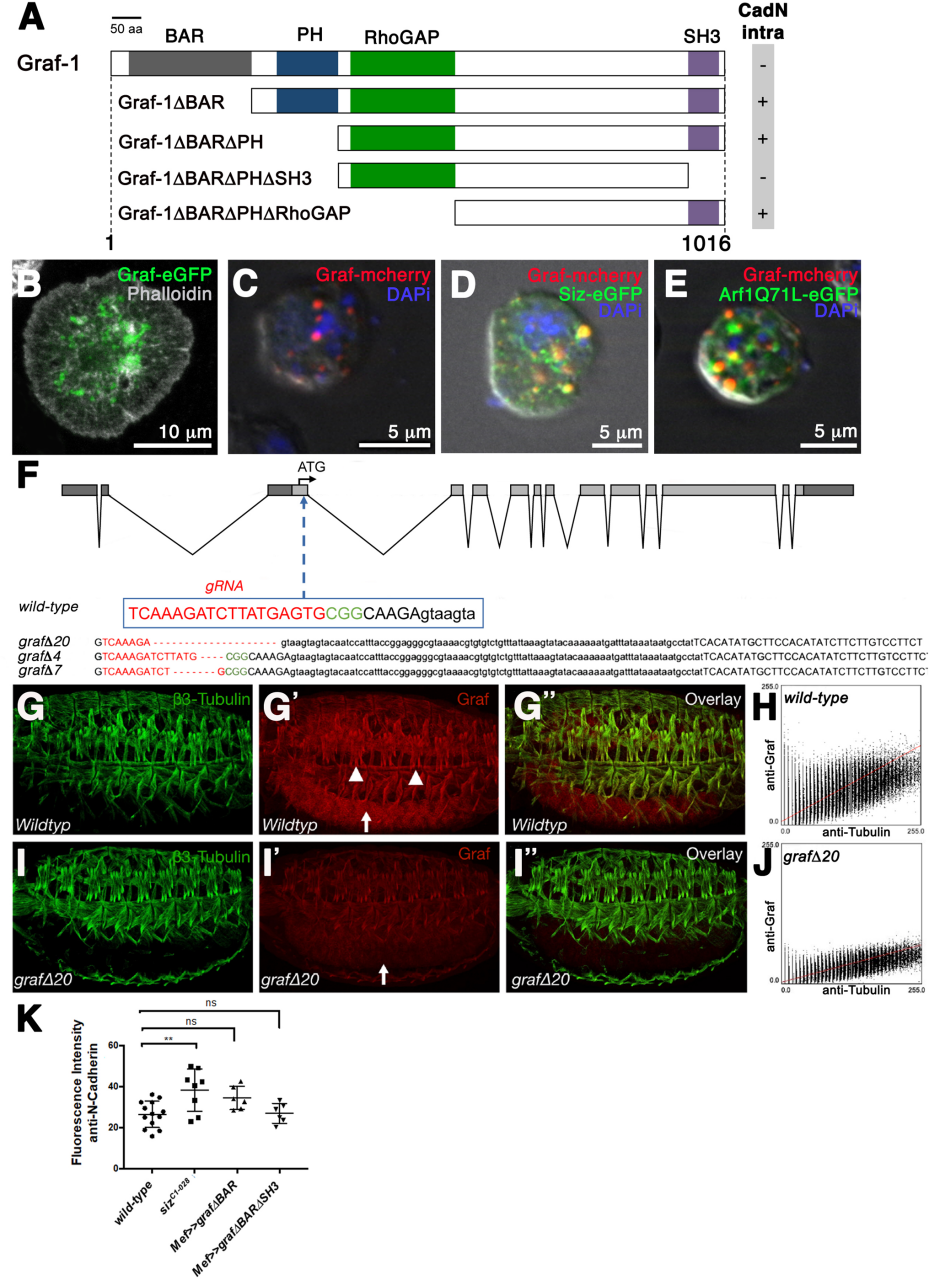
**The *Drosophila* RhoGAP protein Graf-1 interacts with the intracellular domain of N-cadherin, but *graf-1* mutants show no defects in myoblast fusion.** (A) Schematic diagram of full-length and truncated forms of Graf-1 tested for interaction with the intracellular domain of N-cadherin. (B and C) Transfected *Drosophila* S2R+ with UAS-*graf-eGFP* or *UAS-graf-mcherry* and stained with DAPi (blue) and Phalloidin (grey). Cells were plated on concanavalin A coated cover slips (B) and on polylysine coated cover slips (C). Scale bar in B: 10 µm and scale bar in C: 5 µm. (D and E) Transfected *Drosophila* S2R+ with *UAS-graf-mcherry* and UAS-*siz-eGFP* (D) or GTP-bound UAS-*Arf1Q71L* (E). Cells were plated on polylysine coated cover slips and stained with DAPI (blue). Both proteins partially colocalize with Graf-mcherry. Scale bars: 5 µm. (F) Exon intron structure of *graf-1*. The 5 prime untransated and 3 prime untranslated regions are marked in dark grey. The coding region is marked in light grey. The 18-bp target sequence of the gRNA is indicated in red, adjacent to NGG protospacer adjacent motif (PAM) sequence in green. Obtained mutants were analyzed by PCR. Red dashes indicate the identified mutations. (G–G″ and I–I″). Lateral view of stage 16 embryos stained with anti-β3-Tubulin (G and I) and anti-Graf (G′ and I′). (G) Wild-type embryo. (I) Homozygous *graf*Δ*20* mutant embryo. (H) Cytofluorogram of the embryo imaged in G and G′ showing the concentration of β3-Tubulin on the x-axis and Graf on the y-axis. (J) Cytofluorogram of the embryo imaged in I and I′ showing the concentration of β3-Tubulin on the x-axis and Graf on the y-axis. (K) Quantification of the amounts of N-cadherin in wild-type, homozygous *siz^C1-028^* mutant embryos, embryos expressing UAS-*graf*Δ*BAR-eGFP* and UAS*graf*Δ*BAR*Δ*SH3-eGFP* with *Mef-*GAL4. The total fluorescence intensity of 6 to 13 embryos was measured for each experiment. *Mef*-GAL4>>UAS-*graf*Δ*BAR-eGFP* and *Mef*-GAL4>>UAS-*graf*Δ*BAR*Δ*SH3-eGFP* embryos show no significant increase in N-cadherin amounts. Bars represent mean±s.d. *P*-values were calculated using the Dunnett's multiple comparison test. P**<0,0032 compared with wild type; ns, not significant.

Like the mammalian Graf proteins, *Drosophila* Graf-1 contains a BAR, PH, RhoGAP and SH3 domain (Kim et al., 2017) (Fig. 5A). To determine whether *Drosophila* Graf-1 is involved in the removal of N-cadherin, we first performed protein interaction studies between Graf-1 and Schizo and the intracellular domain of N-cadherin. Although we did not detect any interaction between Graf-1 full-length and N-cadherin, we found that Graf-1 lacking the BAR domain interacted with the intracellular domain of N-cadherin (Fig. 5A, Table 1, Fig. S2). The BAR domain of mammalian Graf-1 has been shown to directly interact with the GAP domain to inhibit its activity (Eberth et al., 2009). We assume that the failed interaction between Graf-1 full-length and the intracellular domain of N-cadherin is caused by the autoinhibited conformation of Graf-1. Domain mapping experiments revealed that the SH3 domain of Graf-1 is responsible for the interaction of GrafΔBAR with the intracellular domain of N-cadherin (Fig. 5A, Fig. S3). However, we found no protein interactions between Graf-1 and Schizo in the yeast-two hybrid assay. Subcellular localization assays revealed that Graf-1-eGFP is distributed in a punctuated manner in *Drosophila* S2R+ cells (Fig. 5B and C). Furthermore, we found a partial colocalization between Graf-1, Siz and GTP-bound Arf1 (Fig. 5D and E). To determine whether Graf is involved in the removal of N-cadherin, we used the CRISPR/Cas9 strategy to generate *graf* mutants. Fig. 5F shows the localization of the guide target (gRNA) within the open reading frame of the *graf-1* gene which spans a genomic region of 8.8 kb. The gRNA is located in the third exon of *graf-1* which encodes for the BAR domain. The largest deletion we observed comprises 20 base pairs and was termed *grafΔ20*. The deletion in *grafΔ20* leads to a seven amino acid truncation in the BAR domain of Graf-1, which should result in a premature stop codon after 64 amino acid. To investigate whether the Graf-1 protein is still detectable in *grafΔ20* mutants we employed the Graf-1 antibody from Kim et al. (2017) and performed cytofluorograms of single embryos. The Graf-1 antibody detects a peptide sequence located between the RhoGAP and the SH3 domain. In wild-type embryos Graf-1 is expressed in muscles (Fig. 5G′, arrowhead) and the central nervous system (Fig. 5G′, arrow). In the *grafΔ20* deletion we observed a reduced expression of Graf-1 in muscles (Fig. 5I′). When we analyzed Graf-1 protein levels in comparison to β3-Tubulin, we found that the Graf protein is reduced in *grafΔ20* mutants (Fig. 5H and J). Sequence analyses revealed an alternative open reading frame for Graf-1, which might explain the reduced protein levels. Homozygous *grafΔ20* mutants were viable and showed no defects in myoblast fusion (Fig. 5G and I). In Kim et al. (2017) a *graf*-null mutant was generated by imprecise P-element excision. Homozygous *graf*-null mutants are viable and fertile. In addition, we quantified amounts of N-cadherin in wild-type embryos expressing UAS-*grafΔBAR* and UAS-*grafΔBARΔSH3* with *Mef*-GAL4 (Fig. 5K). However, we failed to observe increased amounts of N-cadherin. Based on these findings, we propose that Graf-1-dependent endocytosis does not play a major role in the removal of N-cadherin from the plasma membrane.

**Table 1.**
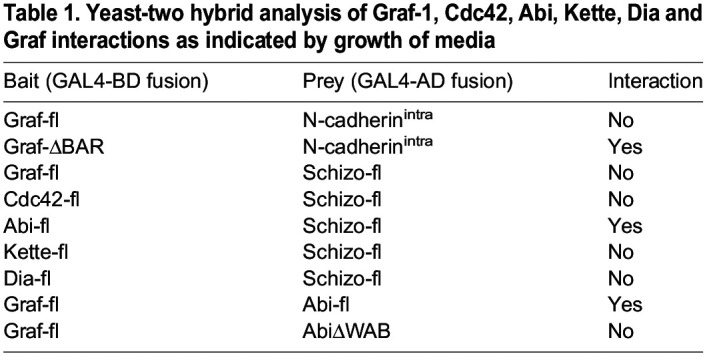
Yeast-two hybrid analysis of Graf-1, Cdc42, Abi, Kette, Dia and Graf interactions as indicated by growth of media

**Fig. 6.**
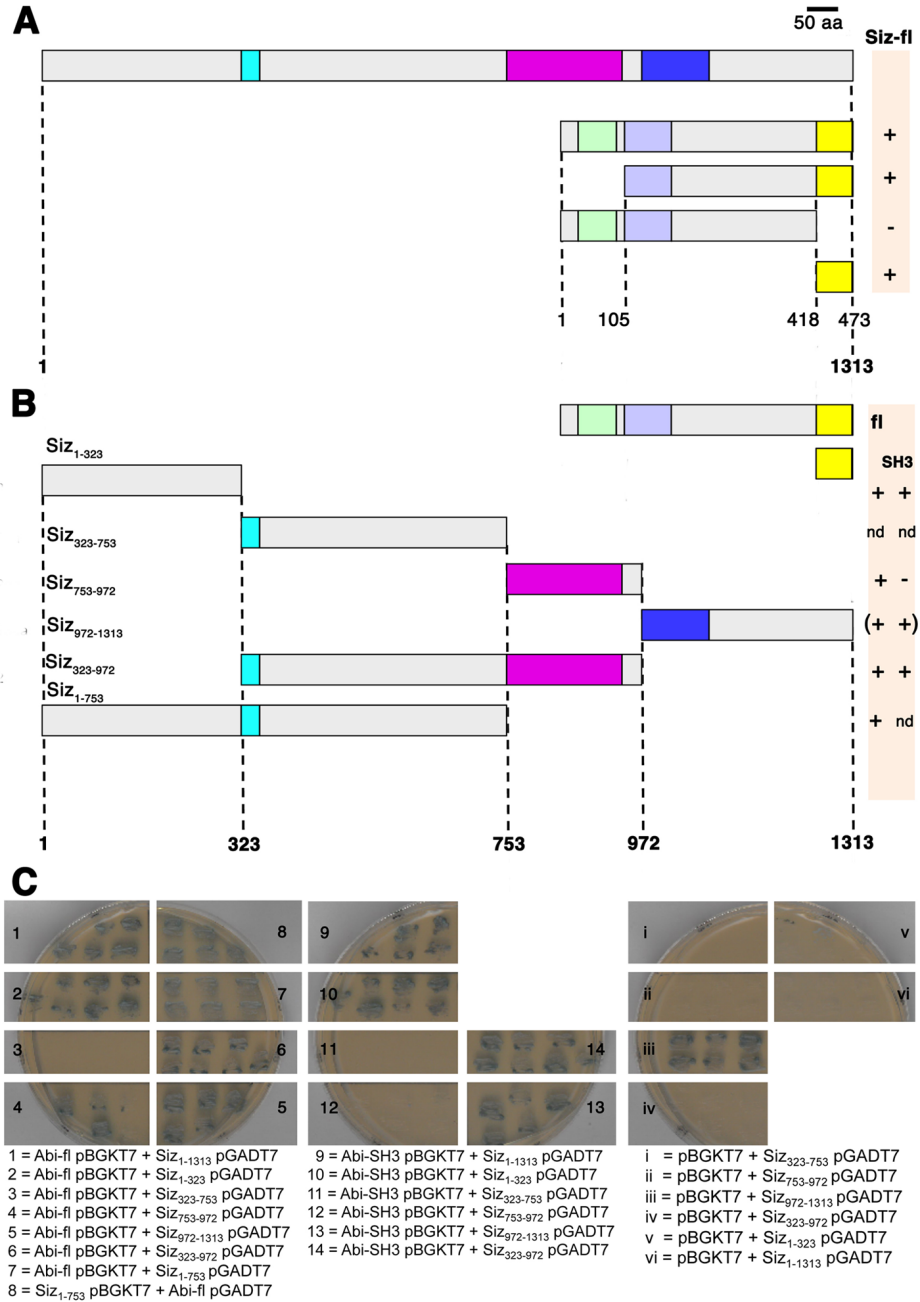
**Abi interacts with the N-terminal region of Schizo via its SH3 domain.** (A) Schematic representation of Schizo and Abi deletions employed to study protein interactions. (B) Schematic representation of Abi and Schizo deletions employed to study protein interactions. (C) Yeast-two hybrid tests. As indicators for interaction the growth on SD-Ade/-Leu/-His/-Trp plates and the implementation of α-Galaktosidase by the Galactosidase MEL1 were used. Plates were incubated for 48 h; -, no growth and blue colour in 48 h; +, growth and blue colour in 48 h. In 1 to 7 Abi full-length cloned into the bait vector pBKGT7 from Clontech was tested for protein interaction with Schizo deletions cloned into the prey vector pGADT7. Schizo full-length Siz_1-1313_, Siz_1-323_, Siz_753-972_, Siz_972-1313_ and Siz_323-972_ showed an interaction with Abi. Control experiments (i–vi) revealed an interaction of Siz_972-1313_ pGADT7 with the empty pBGKT7. In 8 the N-terminal region of Siz_1-753_ cloned into the pBGKT7 interacted with Abi full-length cloned into the pGADT7. In 9 to 14 only the SH3 domain of Abi into the pBGKT7 was used for interaction studies with Schizo truncations. An interaction with Siz_1-1313_, Siz_1-323_, Siz_972-1313_ and Siz_323-972_ was observed.

The actin polymerization machinery has been implicated to aid in clathrin-independent endocytosis (Chadda et al., 2007; Römer et al., 2010; Sathe et al., 2018). Indeed, actin polymerization seems to be a key step to power local membrane deformation and carrier budding in clathrin-independent endocytosis (Hinze and Boucrot, 2018). Since Arp2/3- and Formin-dependent F-actin polymerization is essential for the fusion of myoblasts (Kim et al., 2007; Massarwa et al., 2007; Richardson et al., 2007; Schäfer et al., 2007; Berger et al., 2008; Deng et al., 2015), we determined whether members of the Arp2/3 activation machinery or the formin Diaphanous interact with Schizo (Table 1). Surprisingly, we identified that Abi, a component of the Scar/WAVE complex, interacts with Schizo and Graf-1 (Table 1, Fig. 6 and Fig. S4).

### The SH3 domain of Abi interacts with the N-terminal region of Schizo

We next mapped the interaction domain on Abi by testing deletion mutants of Abi for binding to full-length Siz_1-1313_. Full-length Abi and the deletion mutant proteins examined are depicted schematically in Fig. 6A. The deletion of the carboxy-terminal SH3 domain eliminated the interaction with Schizo Siz_1-1313_ (Fig. 6A). Subsequently, we performed the converse experiment and only used the SH3 domain of Abi for determining the interaction with Schizo (Fig. 6A and C). These data indicate that the SH3 domain is mandatory for the observed interaction. To identify the interaction domain on Schizo, we used the deletion mutant proteins illustrated in Fig. 6B. Abi full-length interacts with Siz_1-323_, Siz_753-972_, Siz_972-1313_ and Siz_1-753_. As demonstrated in the controls, Siz_972-1313_ shows a false positive interaction with the empty pBGKT7 vector. By using the SH3 domain of Abi as bait protein, we could confirm the interaction of Siz_1-323_, Siz_972-1313_ and Siz_1-753_, but not with Siz_753-972_. From these data, we concluded that the N-terminal region of Schizo from 1–753 amino acid is responsible for interacting with Abi. Because of the observed protein interaction, we next transfected *Drosophila* S2R+ cells with UAS-*siz-mcherry* and UAS-*abi-eGFP* to determine the distribution of both proteins in *Drosophila* S2R+ cells. We found that UAS-*abi-eGFP* colocalizes with full-length UAS-*schizo-mcherry* and that both proteins are distributed in a punctuated manner (Fig. S5 and Movie 2).

### Abi and Schizo serve antagonistic functions

**Fig. 7.**
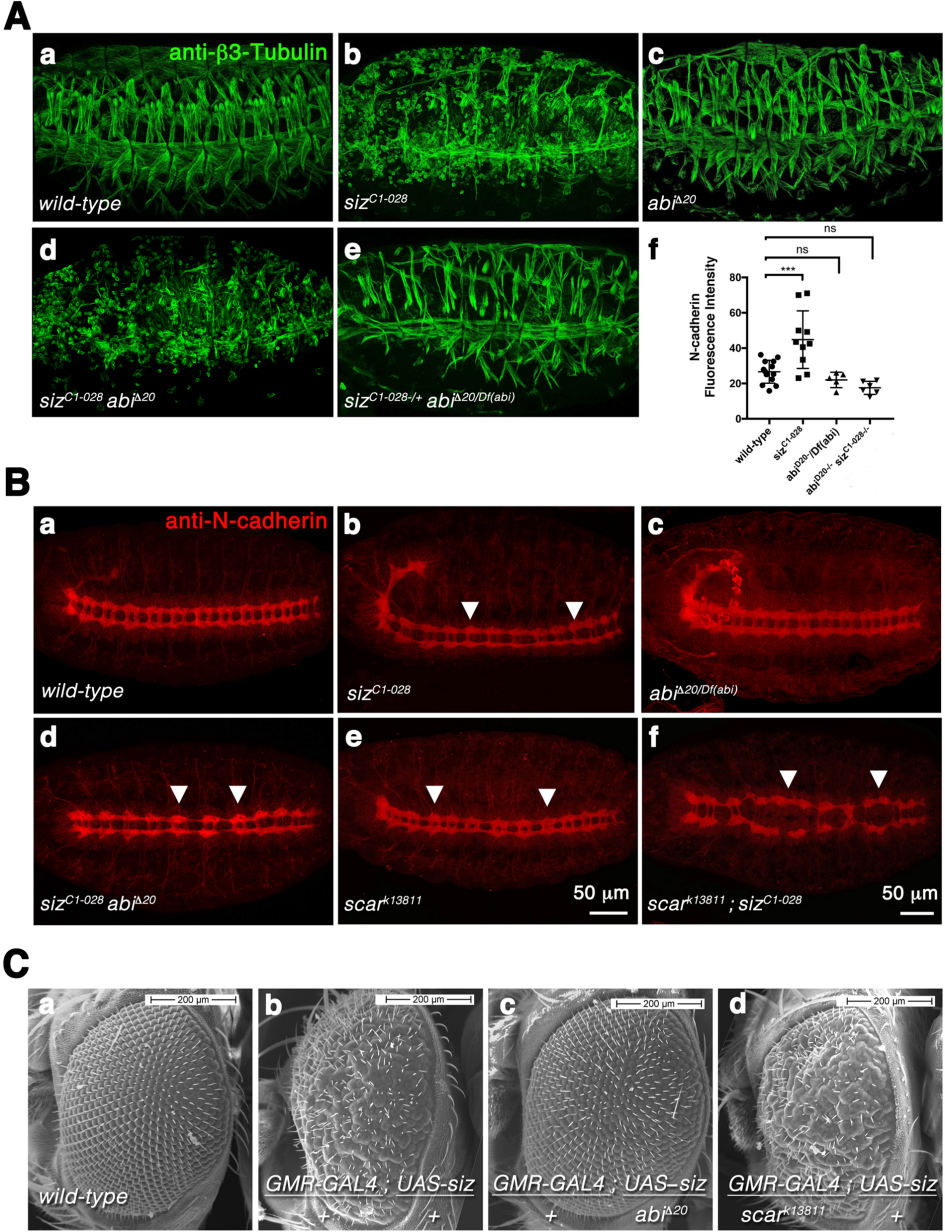
**Abi and Schizo serve antagonistic functions.** (Aa–e) *Drosophila* stage 16 embryos stained with anti-β3-Tubulin. Anterior is left, lateral view. (a) Wild-type embryo. (b) Homozygous *schizo^C1-028^* (*siz^C1-028^*)-mutant embryo. (c) Transheterozygous *abi*^Δ^*^20^*/Df(*abi*)-mutant embryo. (d) Homozygous *abi*^Δ^*^20^ siz^C1-028^* double-mutant embryo. (e) Transheterozygous *abi*^Δ^*^20^*/Df(*abi*) mutant-embryo lacking on copy of *siz^C1-028^*. The muscle pattern of the homozygous *abi*^Δ^*^20^ siz^C1-028^* mutant embryo looks like the muscle pattern of homozygous *siz^C1-028^* mutants. The reduction of *schizo* in *abi*^Δ^*^20^*/Df(*abi*) mutants did not enhance the muscle phenotype of *abi*^Δ^*^20^*/Df(*abi*) mutants. (f) Quantification of amounts of N-cadherin in wild-type, homozygous *siz^C1-028^* and *abi*^Δ^*^20^*mutants and homozygous *abi*^Δ^*^20^ siz^C1-028^* double mutants. The total fluorescence intensity of 5 to 12 embryos was measured for each experiment. Bars represent mean±s.d.; *P*-values were calculated using the Dunnett's multiple comparison test. P***<0,0006 compared with wild type; ns, not significant. (Ba–f) Ventral view of stage 16 embryos stained with anti-N-cadherin in red to visualize commissures of the CNS. (a) Wild-type embryo. (b) Homozygous *siz^C1-028^* mutant embryo with missing posterior commissures (arrows). (c) Transheterozygous *abi*^Δ^*^20^*/Df(*abi*)-mutant embryo exhibited no commissural defects. (d) Homozygous *abi*^Δ^*^20^ siz^C1-028^* double-mutant embryo displays abnormal commissural axon crossing (arrows). (e) Homozygous *scar^k13811^* mutant embryo also displays abnormal crossing of commissural axons (arrows). (f) Homozygous *scar^k13811^ siz^C1-028^* double-mutant embryo shows an enhancement of the *schizo* mutant phenotype. In the double mutant anterior and posterior commissural axons fail to cross the midline. (Ca-d) Scanning electron micrographs of adult eyes. (Ca) Wild-type photoreceptor cells. (Cb–d) Modification of the Schizo overexpression phenotype. (b) The overexpression of UAS-*schizo* in photoreceptor cells with *GMR*-GAL4 induces a rough eye phenotype (*GMR*-GAL4/+; UAS-*siz*/+). (c) This phenotype can be suppressed by taking out one copy of *abi*^Δ^*^20^* (*GMR*-GAL4/+; UAS-*siz*/ *abi*^Δ^*^20^*). (d) Taking out one copy of *scar^k13811^* does not suppress the *GMR*-GAL4/+>>UAS*-siz/+*-induced phenotype.

The observation that Abi and Schizo colocalizes in a punctuated manner raises the question whether Abi and Schizo act in concert to regulate amounts of N-cadherin by endocytosis. To determine whether both proteins contribute to the same process, we performed epistasis experiments and used meitotic recombination to generate *schizo abi*-double mutants. In a first attempt we analyzed the muscle phenotype of the hypomorphic *schizo* allele *siz^C1-028^*, the *abi*-null allele *abi*^Δ^*^20^* and *siz^C1-028^ abi*^Δ^*^20^*-double mutants (Fig. 7Aa–e). We observed that homozygous *siz^C1-028^ abi*^Δ^*^20^* double mutants show a strong myoblast fusion phenotype like *siz^C1-028^* (Fig. 7Ad and Ab). The muscle phenotype of *abi*^Δ^*^20^*-null mutants is comparable to the wild-type muscle pattern (Fig. 7Ac and Aa), but some muscles are missing. If *schizo* and *abi* both contribute to regulation of N-cadherin, we expected to enhance the *abi* muscle phenotype by reducing the *schizo* gene dose. However, the musculature of transheterozygous *abi*^Δ^*^20^/Df(abi)* mutants lacking one copy of *schizo* looks like the musculature of homozygous *abi*^Δ^*^20^* mutant embryos (Fig. 7Ae and Ac). From the phenotypes, we cannot conclude whether *schizo* and *abi* contribute together to regulating N-cadherin. Therefore, we have determined amounts of N-cadherin in homozygous *siz^C1-028^*, *abi*^Δ^*^20^/Df(abi)* single and *siz^C1-028^ abi*^Δ^*^20^* double mutants. Fig. 7Af shows the measurement of the N-cadherin fluorescence intensity from 5 to 12 embryos. Interestingly, we found that amounts of N-cadherin are reduced to wild-type levels in homozygous *siz^C1-028^ abi*^Δ^*^20^* double-mutant embryos. The reduced amounts of N-cadherin in the double mutants indicate that *schizo* and *abi* might act antagonistically. Further support for this notion comes from the analyses of the central nervous phenotype with anti-N-cadherin (Fig. 7Ba–f). N-cadherin is not only expressed in the mesoderm and during myoblast fusion, but also shows a strong expression in the ventral nerve cord (Iwai et al., 1997). When we imaged *siz^C1-028^ abi*^Δ^*^20^* double mutants to determine the fluorescence intensity of N-cadherin, we noticed that the commissural phenotype of homozygous *schizo* mutants (Fig. 7Bb arrowheads) is suppressed in some hemisegments of *siz^C1-028^ abi*^Δ^*^20^* double mutants (Fig. 7Bd arrowheads).

Abi is a member of the Scar/WAVE complex, which is required for the activation of the Arp2/3 complex that nucleates branched F-actin filaments (Rotty et al., 2013). The nucleation ability of the Arp2/3 complex depends on nucleation promoting factors of the WASp family to which Scar/WAVE belongs to (Tyler et al., 2016). Besides its function as member of the Scar/WAVE complex, Abi is also known to interact physically with WASp (Bogdan and Klämbt, 2003). The finding that *abi* antagonizes *schizo* during myoblast fusion and commissural formation suggests that also other members of the Scar/WAVE complex, e.g. *scar/wave* counteract *schizo*. To test this assumption, we generated *scar siz* double mutants by using the hypomorphic *scar* allele *scar^k13811^*. Commissures are reduced in the *siz* alleles *siz^C1-028^* (Figs 2B″ and 7Bb arrowheads). In homozygous *scar^k13811^* mutants, commissures are not reduced, but both commissures are sometimes observed in close proximity (Fig. 7Be arrowheads). In contrast, we found that the commissural phenotype of homozygous *siz^C1-028^* mutant embryos is clearly enhanced in homozygous *scar^k13811^ siz^C1-028^* double-mutant embryos (Fig. 7Bf, arrowheads). Taken together, these data suggest that only *abi* counteracts *schizo*, but not *scar/wave*.

In *Drosophila* about 60% of the genome is maternally contributed as mRNA to ensure embryonic development (De Renzis et al., 2007; Lécuyer et al., 2007). The mRNA of *abi* and *scar* are maternally transcribed (Zallen et al., 2002; Lin et al., 2009). We found that the *schizo* mRNA is still detectable in the *schizo* deficiency Df(3L)*ME178* at embryonic stage 11, when myoblasts start to fuse and at stage 14 (Fig. S6). This suggests that during embryogenesis, reduced protein levels of Abi are required to antagonize Schizo function, but not Scar/WAVE. To support this notion, we overexpressed Schizo in photoreceptor cells in adult flies that have no maternal mRNA using the eye-specific *GMR*-GAL4 driver line. Expression of UAS-*siz* leads to a rough-eye phenotype (Fig. 7Cb). This rough-eye phenotype is suppressed in flies heterozygous for the *abi*^Δ^*^20^* mutation (Fig. 7Cc). Consistently with our previous results, the rough-eye phenotype is not suppressed in flies heterozygous for the hypomorphic *scar* allele *scar^k13811^* (Fig. 7Cd). In addition, we observed no suppression of the rough-eye phenotype in flies heterozygous for the *rac* null allele *rac1^J11^*, which is another member of the Scar/WAVE complex (Fig. S7B). Moreover, we examined whether the Abi interacting partner WASp is able to antagonize Schizo function. However, we found that the reduction of the *wasp* dosage using the dominant-negative EMS allele *wasp^3D3-035^* enhances the *GMR-*GAL4>>UAS-*siz* induced rough-eye phenotype (Fig. S7C). In summary, these experiments confirm our hypothesis that only reduced levels of Abi antagonize Schizo function.

## DISCUSSION

Understanding the multiple regulatory layers of GEF activation that allows coordinating the GDP/GTP exchange on small GTPases is an important issue. To date, the multitude of molecular interactions leading to GEF activation are most advanced for the Ras activator Son of Sevenless (Bandaru et al., 2019). Studies on the subfamily of Arf GEFs that carry a PH domain associated with a catalytic Sec7 domain have concentrated on the interaction of the GEF with Arf GTPases and phospholipids. Membrane recruitment of Arf GTPases is mediated by a myristoylated N-terminal amphipathic helix (Franco et al., 1996; Goldberg, 1998; Liu et al., 2009) and is crucial for the activation by the GEF (Pasqualato et al., 2001; Randazzo et al., 1995). The PH domain binds phophatidyl inositol 3,4,5-triphosphate (PIP3) and phosphatidyl insositol 4,5-bisphosphate (PIP2) (Chardin et al., 1996; Kavran et al., 1998; Klarlund et al., 2000). In structural studies with myrArf/BRAG2 bound to a PIP2-containing bilayer, the myristoylated N-terminal helix of Arf is close to the Sec7 domain and it has been proposed that the Sec7 domain might recognize conformational information from the amphipathic helix (Karandur et al., 2017). However, so far, the influence of receptor binding for Arf GEF activation has not been taken into account.

In this study, we found that N-cadherin levels were elevated in mutants of the Arf1 GEF *schizo* and reduced in embryos expressing only the Sec7-PH domain of Schizo. These findings are in line with studies on mammalian GEP100/BRAG2. The siRNA-mediated depletion of GEP100/BRAG2 in HepG2 cells resulted in increased E-cadherin content (Hiroi et al., 2006). Furthermore, increased amounts of β1-integrin were observed in the ‘knock-down’ of BRAG2 in HeLa cells and it was proposed that BRAG2 served specifically for β1-integrin internalization (Dunphy et al., 2006). Rescue experiments with the Sec7-PH domain and overexpression studies further support the notion that it acts as a constitutively active form of Schizo during myoblast fusion.

The finding that the member of the BRAG2 subfamily Schizo interacts with N-cadherin via its N-terminal region let us investigate the importance of this region for Schizo function. Our data imply that the N-terminal region provides additional layers of GEF regulation. First, we identified that Schizo undergoes nucleocytoplasmic shuttling in the absence of the N-terminal region. A nuclear localization for mammalian BRAG2a and BRAG2b was also detected in HeLa and MDCK cells after overexpression (Dunphy et al., 2006). BRAG2 possesses like Schizo a nuclear localization sequence in the Sec7 domain. However, the finding that the expression of Siz_753-1081_ partially rescues the *schizo* mutant phenotype let suggest that nuclear localization does not disturb Schizo function. Furthermore, the overexpression of Siz_753-1081_ does not result in myoblast fusion defects as observed for Siz_753-1313_ suggesting that it does not act as a constitutive-active form. Second, we found that Abi binds to the N-terminal region of Schizo. Abi has been reported to regulate actin polymerization by formation of complexes with Scar/WAVE and WASp (Innocenti et al., 2005). Furthermore, it has been described to modulate EGFR endocytosis (Tanos and Pendergast, 2007). Besides, Abi-1 has been identified to interact with the Ras activator Son of Sevenless (Scita et al., 1999; Fan and Goff, 2000).

Vesicle scission in Clathrin-independent endocytosis depends on specialized actin-based platforms (Doherty and McMahon, 2009; Mayor et al., 2014). However, there is no unifying theme and multiple mechanisms may co-exist. The molecular machinery of CLIC/GEEC-dependent endocytosis involves the activation of Arf1 by the Arf GEF GBF1 and the RhoGAP protein Graf that removes Cdc42 from the plasma membrane (Kumari and Mayor, 2008; Gupta et al., 2009). A recent study has addressed the spatiotemporal localization of known molecules affecting CLIC/GEEC endocytosis by using real-time TIRF microscopy (Sathe et al., 2018). In this study it was reported that Arp3 recruitment occurred earlier to endocytic vesicles than Cdc42. Furthermore, N-WASp failed to recruit to form CLIC/GEEC endocytotic sites. These data imply alternative pathways for Arp2/3 activation in CLIC/GEEC endocytosis. In *Drosophila*, WASp lacking the Cdc42-binding domain (WaspΔCRIB) is still able to rescue the adult phenotype of *wasp* mutants and it was proposed that other elements than Cdc42 contribute to *Drosophila* WASp activation (Tal et al., 2002). Such an alternative element might be Abi. However, our genetic interaction studies suggested that Abi counteracts Schizo function.

Dissecting the function of Arp2/3-dependent endocytosis during myoblast fusion is challenging since the fusion of myoblasts depends on Scar/WAVE- and WASp-dependent Arp2/3 activation (Kim et al., 2007; Massarwa et al., 2007; Richardson et al., 2007; Schäfer et al., 2007; Berger et al., 2008). This might explain why the myoblast fusion phenotype in *abi siz* double mutants is not suppressed although amounts of N-cadherins are reduced. Therefore, we overexpressed Schizo in photoreceptor cells and performed gene dose experiments and found that only the reduction of *abi* suppressed the Schizo-induced overexpression phenotype.

In *Salmonella* host cell invasion, the Arf6 GTPase was shown to affect indirectly actin polymerization by activating the GEF ARNO (Humphreys et al., 2013). Arf6 is activated by EFA6 or BRAG and recruits the autoinhibited GEF ARNO to the plasma membrane. The autoinhibition of ARNO is released by the binding of activated Arf6 to its PH domain. As a consequence, ARNO activates Arf1, which induces together with Rac1 the activation of the WAVE Regulatory Complex (Koronakis et al., 2011; Humphreys et al., 2013). Since we found no evidence that *scar/wave* or *wasp* exert a regulatory influence on Schizo, we propose that Abi might be part of a dosage-dependent regulatory feedback mechanism following Arp2/3-dependent actin polymerization.

**Fig. 8.**
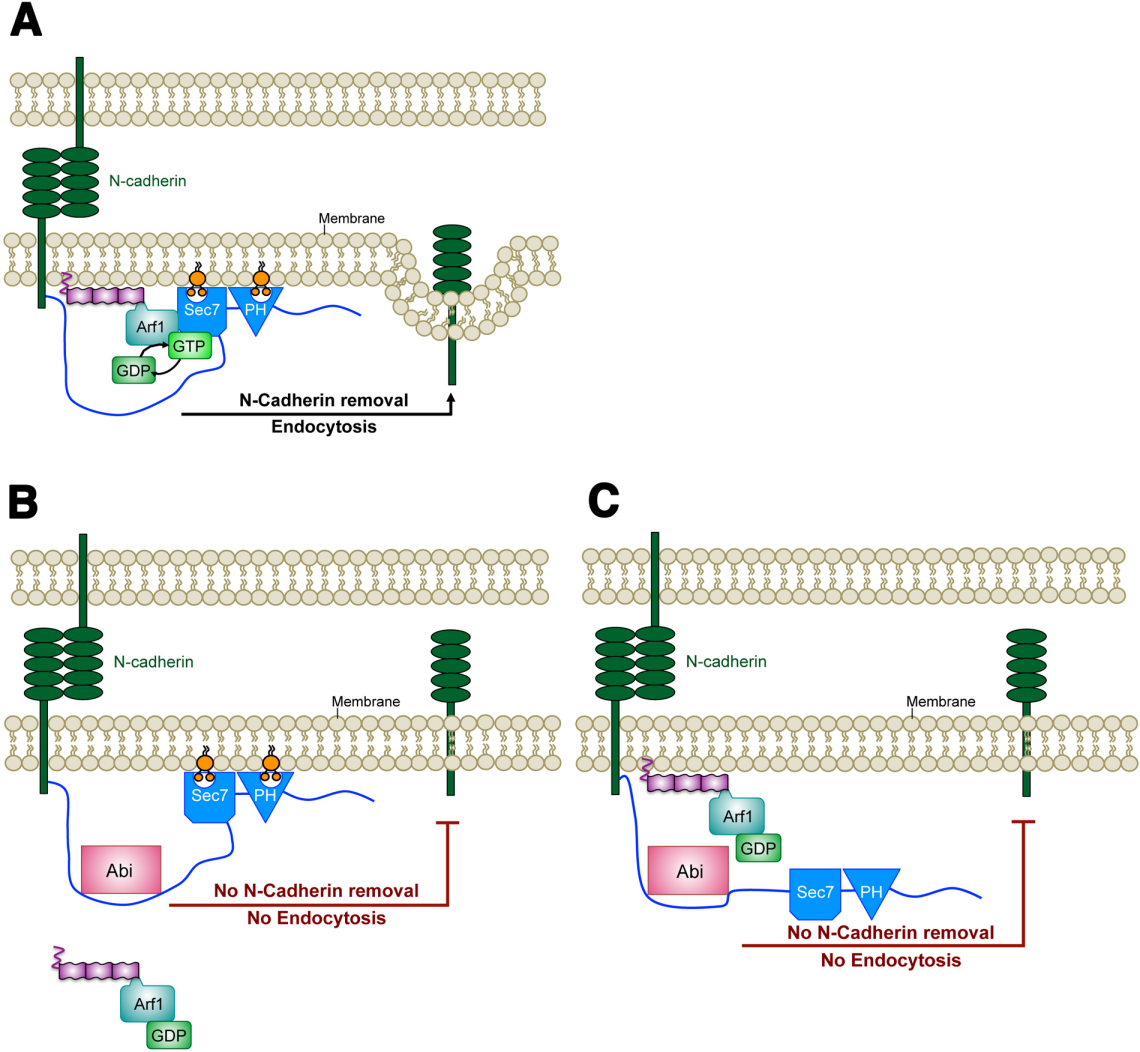
**Contingent models indicating how the binding of Abi might antagonize Schizo function.** (A) The N-terminal region of Schizo interacts with the intracellular region of N-cadherin to regulate amounts of N-cadherin. The activity of Schizo involves the interaction of the Sec7 domain with the Arf1-GTPase and the lipid interaction of the Sec7 and PH domain. This induces the removal of N-cadherin. (B and C) The binding of Abi to the N-terminal region of Schizo counteracts N-cadherin regulation in a dosage-dependent manner. (B) The binding of Abi to the N-terminal region of Schizo could prevent the binding of the myristoylated Arf-GTPase to Schizo, which inhibits the removal of N-cadherin. (C) Alternatively, Abi might prevent the lipid interaction of the Sec7 and PH domain thereby antagonizing Schizo function.

The data presented in this study point towards a novel model for the regulation of the Arf1 GEF Schizo (Fig. 8). First, we suggest that amounts of N-cadherin are regulated by the Sec7 GEF Schizo and that the N-terminal region of Schizo is crucial for the regulation of its activity (Fig. 8A). The Abl-interacting partner Abi might compete with N-cadherin for Schizo binding and disturbs the removal of N-cadherin. However, since the expression of the constitutive-active form of UAS-Sec7-PH decreases amounts of N-cadherin in the absence of the N-terminal region, we propose that Abi acts rather as an allosteric modulator by either inhibiting the binding of myristyolated Arf1 (Fig. 8B) or by inhibiting the binding of the Sec7-PH module to the lipid bilayer (Fig. 8C). A second regulatory mechanism to control Schizo activity might involve the nuclear localization sequence within the GEF domain in the absence of the N-terminal region. The data reported in our study will be valuable for future structural analyses to determine how Abi prevents GEF activity.

## MATERIAL AND METHODS

### *Drosophila* stocks and genetics

Generation of pUASt-attB-eGFP-siz1-1313, pUASt-attB-eGFP-siz753-1313 flies and siz-Sec-PH

The *siz1-1313* and *siz753-1313* construct were amplified from the *LP01489* cDNA by PCR, subcloned into the pENTRTM/D-TOPO vector and recombined into the Gateway vector pUASt-attB-rfa-eGFP. We used the following primer pairs:

siz753-1313_f: 5′-CACCATGGAGACGATACGCAAG-3′

siz753-1313_rev: 5′-TTAGACCTCCGTCGACCGT-3′

sizSec7-PH_f: 5′-CACCATGTCGGAGAC-3′

sizSec7-PH_rev: 5′-GAGATGGAGTCGTGA-3′

#### graf mutant flies

*y1cho2v1*; *graf* mutants were generated using a single target construct in the CRISPR/Cas9 system as described by Kondo and Ueda (2013). For cloning of the target construct, primers 5′-CTTCGGTCAAAGATCTTATGAGTG-3′ and 5′-AAACCACTCATAAGATCTTTGACC-3′ were used, and the product was ligated to *pBFv-U6*.*2*. The target construct was injected into *y2cho2v1P{nos-phiC31\int*.*NLS}X;attP2(III)*, and established transgenic flies were crossed with *y2cho2v1*;*attP40{nos-Cas9}/CyO* flies for mutagenesis. Twenty-three founder flies were crossed with *y2cho2v1*; *Sco/CyO* flies to establish potential mutants. Genomic regions next to the target sequence of homozygous offspring were analyzed by PCR and screened for deletions. We identified three different deletions and an additional sequence insertion.

#### Generation of pUASt-graf-ΔBAR and pUASt-ΔBARΔSH3

The graf-ΔBAR and pUASt-ΔBARΔSH3 constructs were amplified from the full-length *graf* LD28528 cDNA obtained from DGRC. It should be noted that the cDNA clone reported as fully sequenced in flybase, contains an additional nucleotide at position 467, which leads to a shift of the open reading frame. As a consequence, the deduced protein from this cDNA lacks the BAR domain. To generate full-length Graf, we performed a site-specific mutagenesis on the LD28528 cDNA to remove the additional nucleotide.

#### Generation of germline clones

*abi* germline clones were produced by heat-shock in *hs-Flp; FRT82B ovo^D^/FRT82B abi^Δ20^* larvae. The resulting females were crossed to males of the *abi* deficiency Df(3R)BSC617/TM3-Dfd-lacZ. This allowed the detection of a wild-type parental contribution on the basis of β-galactosidase.

#### Other stocks

The hypomorphic *schizo* EMS allele *siz^C1-028^* was generated by Hummel et al. (1999) and the CNS and muscle phenotype of *siz^C1-028^* was characterized and described in Önel et al. (2004) and Dottermusch-Heidel et al. (2012). UAS-*Arf1T31N-eGFP* was generated and described in Dottermusch-Heidel et al. (2012). *abi^Δ20^* mutants were kindly provided by Sven Bogdan (Department of Molecular Cell Physiology, Institute for Physiology and Pathophysiology, Philipps-Universität Marburg, Germany) (Stephan et al., 2011). The *twist-*GAL4 driver line SG24 was obtained from the Bloomington Stock Center. As *abi* deficiency we used the deficiency line Df(3R)BSC617 (BL25692) from the Bloomington Stock Center. Further GAL4 driver lines that were used in this study are *Mef*-GAL4 (Ranganayakulu et al., 1996) and TGX *twist*-GAL4 (from A. M. Michelson, National Institutes of Health (NIH), National Heart, Lung, and Blood Institute (NHLBI), USA). As blue balancers we used *Dr*/TM3 *Dfd-lacZ* and *If/*CyO *hg-lacZ*. All crosses were performed at 25°C using standard methods.

### Yeast two-hybrid assay

Yeast two-hybrid experiments were carried out using the Matchmaker GAL4 Two-Hybrid System 3 (Takara Clontech) according to the manufacturer's instructions. For construct generation, the *schizo* cDNA LP01489 from DGRC was used. The following primers were used to generated the different *schizo* and *graf* constructs:

siz1-323 5′-CATATGATGTCCAGGTGTGA-3′ and 5′-GGATCCTCGCACTCCGC-3′

siz1-758 5′-CATATGATGTCCAGGTGTGA-3′ and 5′-

siz324-753 5′-CATATGATGGCCCGTAACG-3′ and 5′-GGATCCTATCGTCTCCGAC-3′

siz754-993 5′-CATATGATGCGCAAGCGAC-3′ and 5′-GGATCCCACACCAGGTCG-3′

siz994-1313 5′-CATATGATGCATCAGCGCG-3′ and 5′-GGATCCTTAGACCTCCGTC-3′

siz324-993 5′-GAATTCATGGCCCGTAACGCA-3′ and 5′-CTCGAGCACACCAGGTCG-3′

graf 5′-CATATGATGGGCGGCGGCAAAAAT-3′ and 5′-GGATCCTAATGGT GCGGCTTCAAAT-3′

GrafΔBAR 5′-CATATGATGTCAACTAAAAAGCCCGAA-3′ and 5′-GGATCCCTAATGGTGCGGCTTCAAAT-3′

GrafΔBARΔPH 5′-CATATGCTGGCTCCCGGCA-3′ and 5′- GGATCCCTAATGGTGCGGCTTCAAAT-3′

grafΔBARΔSH3 5′-CATATGATGTCAACTAAAAAGCCCGAA-3′ and 5′-GGATCCGGTGCCCGTTGA-3′

grafΔBARΔPHΔRhoGAP 5′-CATATGAGCGCCGATATCAA-3′ and 5′-GGATCCCTAATGGTGCGGCTTCAAAT-3′

The products were cloned into the pCRII-TOPO^®^ vector (Invitrogen). The bait vector pGADT7 was digested with *Nde*I and *BamH*I. *siz* was cloned with EcoRI and XhoI into the pGADT7.*siz* was cloned with *NdeI* and *EcoRI* into the pGADT7.

The pGADT7-T and pBGKT7-p53 pair was used as a positive control. The candidate interaction pairs were co-transformed into yeast strain AH109, and the transformed yeast cells were selected using synthetic dropout (SD/-Leu/-Trp) medium, and then further selected on SD/-Leu/-Trp-His/-Ade selective medium with X-α-Gal (80 mg/l). Results were obtained after 2 days of growth at 30°C.

### *Drosophila* cell culture

*Drosophila* S2R+ cells obtained from the *Drosophila* Genomic Rescource Center (DGRC) were propagated in 1× Schneider's *Drosophila* medium (Invitrogen) containing 10% fetal bovine serum at 25°C, and transiently transfected described by Kaipa et al. (2013) by using the FuGENE^®^ HD Transfection Reagent (Promega).

### Immunofluorescence

Embryos were fixed and immunohistochemically analyzed as described by Schäfer et al. (2007). The following antibodies were used at the noted dilutions: rat anti-CadN-Ex#8 (Iwai et al., 1997) 1:50 (Developmental Studies Hybridoma Bank), guinea pig anti-β3Tubulin (Buttgereit et al., 1996; Leiss et al., 1988) 1:10,000, rabbit anti-β-Gal 1:5000 (Biotrend), rabbit anti-GFP 1:500 (abcam ab5665). Primary antibodies were detected using the fluorescent labeled antibodies Alexa-Fluor-488, 568- or 647-conjugated anti-guinea pig, anti-rabbit and anti-rat IgG at a dilution of 1:500 (Invitrogen). DNA was stained with Hoechst reagent (5 g/ml; Sigma-Aldrich), and F-actin was stained with Alexa-Fluor-647–phalloidin (1:100, Invitrogen). For all stainings, specimens were embedded in Fluoromount-G™ (Thermo Fisher Scientific) and observed under a Leica TCS Sp2, TCS Sp5 or TCS Sp8 confocal microscope.

### Statistical test

Statistical analyses were performed with GraphPad Prism software (PRISM7). For Figs 4, 5 and 7 significance was determined by one-way ANOVA with comparison.

### Microscopy and image analysis for fluorescence intensity measurements

A Leica TCS Sp2 (Fig. 1B–E″), TCS Sp5 (Fig. 3A–F) and TCS SP8 (Figs 1F–G′, 2, 4, 5 and 7) confocal microscope was used for fluorescence imaging. The same parameter settings were used to image all samples of the same type. Embryos were embedded in Fluoromount-G^TM^ (Thermo Fisher Scientific) and scanned using the 20× objective with the galvo scanner at 400 Hz. For the fluorescence intensity measurement, we used the counting modus of the TCS SP8. Laser intensity for imaging β3-Tubulin stainings with Alexa 488 were always set to 0.8%. Laser intensity for imaging N-cadherin stainings with Alexa 568 were always set to 4%. The raw data of the embryo images were analyzed using the FIJI software (Schindelin et al., 2012). A SUM-stack was generated in Fiji and the mean fluorescence intensity of lateral imaged embryos were measured by manually drawing a circle around the embryo to select the area to be measured. Additionally, three nearby areas were also measured to analyze the background fluorescence level. The corrected total relative intensity of each embryo was calculated as follows:

(Areaembryo×Mean–Intensityembryo) – (Areaembryo×Background)

To raise cytofluorograms of single embryos we imaged embryos as we did for fluorescence intensity measurements. We employed the JACoP plugin to generate the cytofluorograms (Bolte and Cordelières, 2006), which was downloaded from https://imagej.net/plugins/jacop.

For time-lapse imaging, the Spinning disc microscope from Zeiss was used with the 63× objective.

### Protein extracts from embryos for quantitative western blot analysis

Embryos were collected on apple juice plates, dechorionated (using 50% bleach solution in water) and selected under the GFP stereomicroscope from Leica for GFP expression. For each protein extract 10 embryos of stages 15 to 16 were selected and punctured with an inulin syringe. The resulting lysate was mixed with 10 µl of 1x Lammeli buffer (1 µl per embryo lysate) and transferred into an Eppendorf tube as described by Prudêncio and Guilgur (2015). The samples were heated for 5 min at 98°C and subsequently loaded to a 12% SDS-PAGE gel. Following separation, proteins were transferred to a nitrocellulose membrane (Whatman) by semi-dry blotting (Mini PROTEAN Tetra System; Trans-Blot-turbo Transfer System, BIO-RAD). The membrane was blocked in TBST-blocking buffer [50 mM Tris–HCl pH 7.5, 150 mM NaCl and 0.5% (v/v) Tween 20] containing 5% nonfat dried milk for at least 30 min. The following primary antibodies were used: anti-CadN-Ex#8 (1:100), anti-Actin (Millipore MAB1501, 1:2000). The antibodies were removed by washing three times for 10 min each in blocking solution and the secondary antibodies anti-mouse-HRP (sc-516 102, Santa Cruz Biotechnology, 1:5000) and anti-rat-HRP (Thermo Fisher Scientific #31407, 1:10.000) were applied for 1 h at room temperature. Chemiluminescence was detected using SuperSignal West Dura Extended Duration Substrate (Thermo Fisher Scientific) and Odyssey Fc Imaging System (LI-COR Bioscience) and analyzed by Image Studio Software (LI-COR). The quantitative analysis was performed by Image Studio Software (LI-COR) analysis tool. A volume box of same area was drawn around each protein band to measure the signal intensities respectively. The final signal intensity was calculated by subtracting the Median Local background signal. The ratios of signal intensity values for N-Cadherin to Actin for both wild-type and *Mef>>siz-Sec7* PH were calculated and normalized to represent them as a bar graph.

### Scanning electron microscopy

All *GMR-*GAL4>UAS-*siz*-expressing flies were raised at 25°C. Eyes were fixed in 6% glutaraldehyde and 1% formaldehyde in 0.2 M Hepes buffer for 16 h. Samples were then dehydrated in a 25%, 50%, 70% and 96% ethanol series for 12 h each and finally transferred into acetone by three 10-min changes with 100% acetone. The samples were critical-pointdried by using a Polaron E 3000 (Balzers Union). Samples were attached to sample stubs (Plano GmbH) and sputtered with gold under vacuum using a sputter coater (Balzers Union, Lichtenstein). Scanning electron micrographs of adult fly eyes were taken using a Hitachi S-530 SEM.

## Supplementary Material

10.1242/biolopen.058666_sup1 Supplementary information
