# Supplementary material for: The Abl-interactor Abi suppresses the function of the BRAG2 GEF family member Schizo
Source: Biol Open. 2024 Jan 5;13(1):bio058666. doi: 10.1242/bio.058666 (PMC10810563; doi:10.1242/bio.058666)
Supplement: Supplementary information [file biolopen-13-058666-s1.pdf]

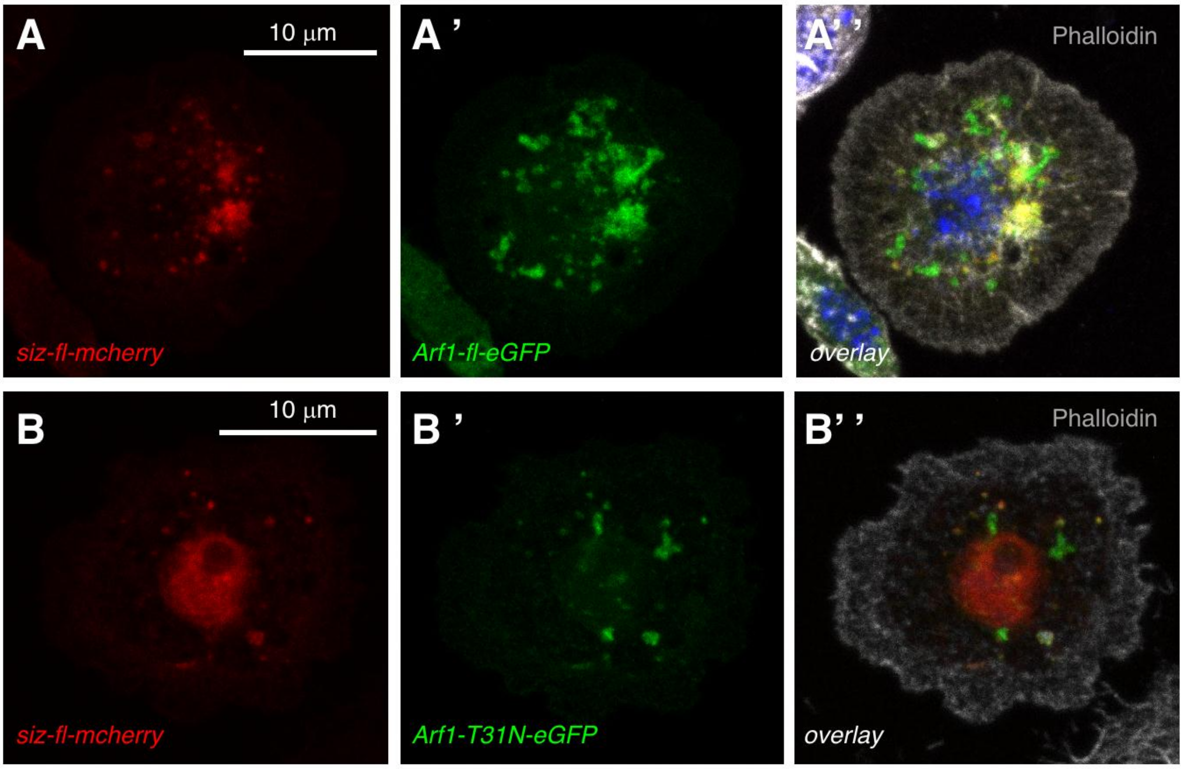

Fig. S1. Arf1 colocalizes with Schizo in *Drosophila* S2R+ cells. (A–A'') Co-transfected *Drosophila* cells with UAS-*siz*$_{1\text{-}1313}$*-mcherry* full-length and UAS-*Arf1-full-length-eGFP*. (A) Siz$_{1\text{-}1313}$ and (A') Arf1-full-length localize in a dot-like pattern and many of these spots co-localize (A''). B–B'' Co-transfected *Drosophila* cells transfected with UAS-*siz*$_{1\text{-}1313}$*-mcherry* full-length and dominant-negative UAS-*Arf1T31N-eGFP*. (B') UAS-*Arf1T31N-eGFP* localizes like UAS-*Arf1-full-length-eGFP* in a dot-like pattern and partially colocalizes with UAS-*siz*$_{1\text{-}1313}$*-mcherry* (B''). Interestingly, we sometimes observe a nuclear localization of UAS-*siz*$_{1\text{-}1313}$*-mcherry* in the presence of dominant-negative Arf1.

Biology Open • Supplementary information

Biology Open (2021): doi:10.1242/bio.058666: Supplementary information

1: AD-Graf-full-length + DBD empty

2: AD empty + DBD-Graf-full-length

3: AD-Graf-ΔBAR + DBD empty

4: AD empty + DBD-Graf-ΔBAR

5: AD-N-cadherin^{intra} + DBD-Graf-full-length

6: AD-Graf-ΔBAR + DBD-N-cadherin^{intra}

7: AD-N-cadherin^{intra} + DBD-Graf-ΔBAR

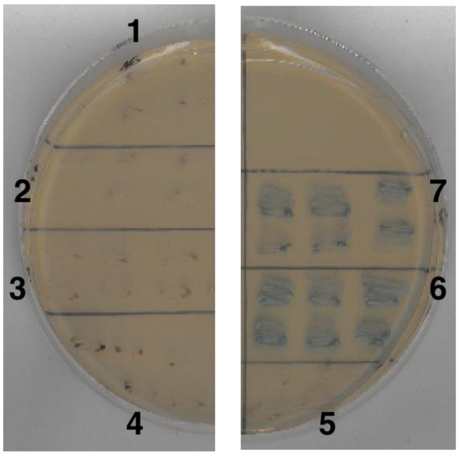

Fig. S2. GrafΔBAR interacts with the intracellular domain of N-cadherin. The full-length Graf cDNA LD28528 from DGRC lacking the BAR domain and the LD28528 cDNA that was modified by site-specific mutagenesis to generate full-length Graf (see Material and Methods) were cloned into the bait vector pBGKT7 (BDB) and tested for interaction with the intracellular domain of N-cadherin. 1–4 show control experiments. No interaction was detected between Graf-full-length and the intracellular domain of N-cadherin (5). However, we observed an interaction between Graf lacking the BAR domain and the intracellular domain of N-cadherin when either GrafΔBAR was used as bait (7) or N-cadherin^{intra} (6).

Biology Open • Supplementary information

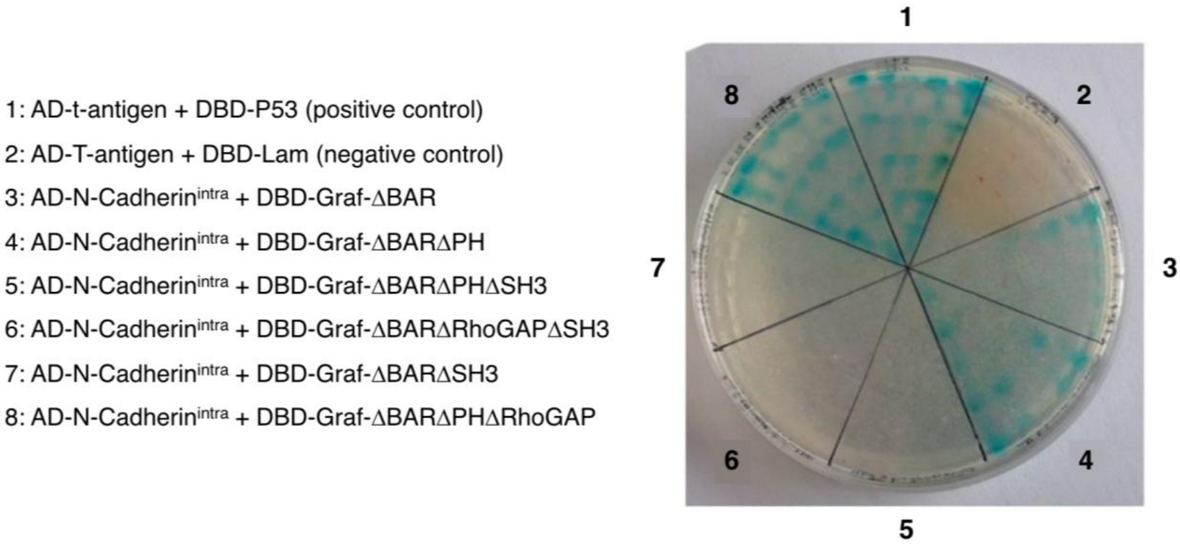

Fig. S3. The SH3 domain of Graf is responsible for the interaction with the intracellular domain of N-cadherin. To map the domain on the side of Graf that interacts with the intracellular domain of N-cadherin, Graf lacking the BAR domain was cloned into the bait vector pBGKT7 (BDB). 1–2 show control experiments. In the absence of the BAR, PH and RhoGAP domain Graf still interacts with the intracellular domain of N-cadherin (3, 4 and 8). Only in the absence of the SH3 domain GrafΔBAR failed to interact with the intracellular domain of N-cadherin (6–7).

Biology Open • Supplementary information

1: AD empty + DBD empty

2: AD-t-antigen + DBD-P53 (positive control)

3: AD-T-antigen + DBD-Lam (negative control)

4: AD-Graf-full-length + DBD-Abi-full-length

5: AD-Abi-full-length + DBD-Graf-full-length

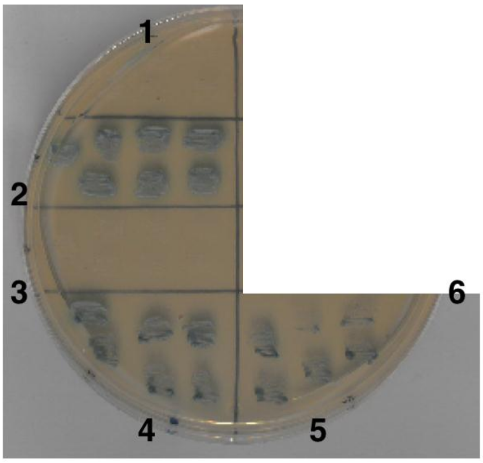

Fig. S4. The Abelson-interactor Abi interacts with *Drosophila* Graf. In an attempt to identify members of the Arp2/3 activation machinery for interaction with Schizo and Graf, we identified Abi as a possible Graf interaction partner. 1–2 show control experiments. In 4 Abi full-length was used as a bait protein. In 5 Graf full-length was used as a bait protein.

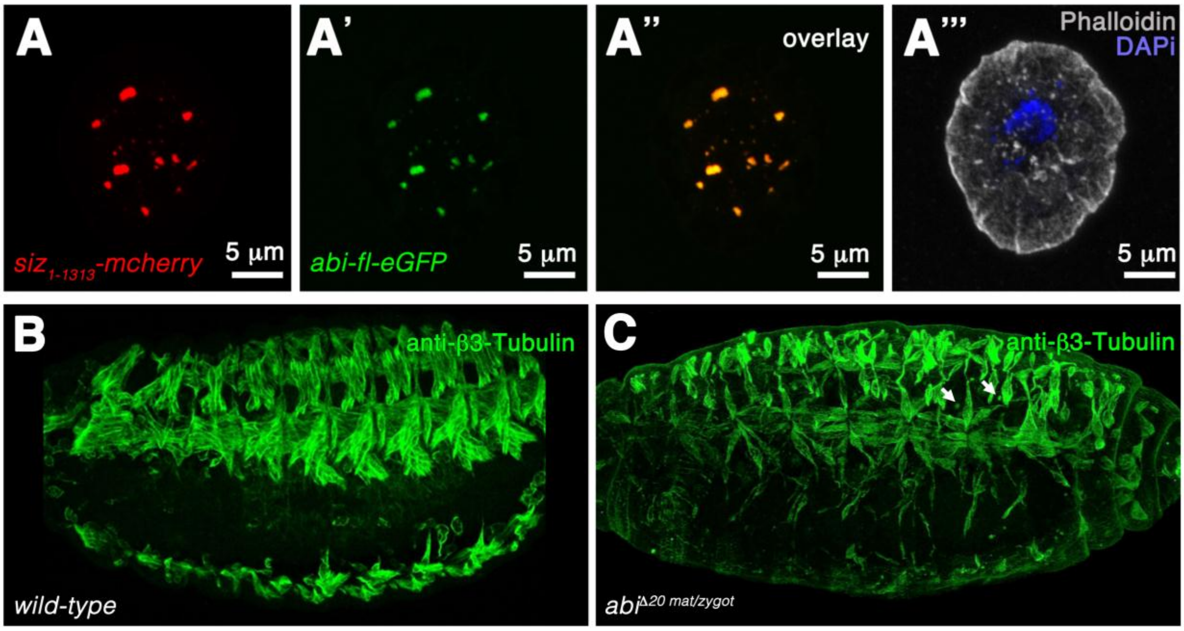

Fig. S5. Colocalization of Schizo and Abi in *Drosophila* cells and removal of the maternal contribution in *abi*$^{\Delta 20}$ zygotic mutants. (A-A''') Co-transfected *Drosophila* S2R+ cells with UAS-*siz-mcherry* (A) and UAS-*abi-eGFP* (A') stained with DAPi (blue) and Phalloidin (grey). Cells were plated on concanavalin A coated cover slips. Scale bar 5 µm. (B) Ventrolateral view of a stage 16 wild-type embryo stained with anti-β3-Tubulin. (C) Ventrolateral view of a stage 16 *abi*$^{\Delta 20\ mat/zygot}$ mutant embryo showing unfused myoblasts (arrows) and severe defects in muscle formation.

Biology Open • Supplementary information

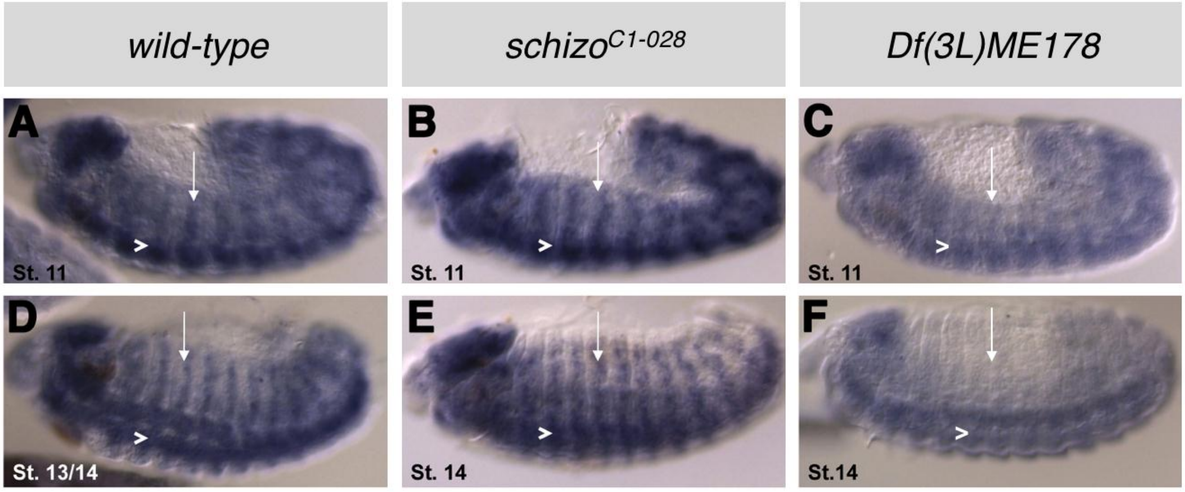

Fig. S6. Distribution of the *schizo* mRNA in wild-type and *schizo*-deficient embryos. (A, D) Wild-type embryos. B, E Homozygous *schizo*$^{C1\text{-}028}$ mutant embryos. (C, F) *schizo*-deficient embryos that are homozygous for the Df(3L) ME178 deficiency (78A7-B1; het). (A, D) The *schizo* mRNA is transcribed at stage 11 (A) and at stage 14 (D) in the somatic mesoderm during myoblast fusion (arrow) and in the central nervous system (arrowhead). (B, E) In homozygous *schizo*$^{C1\text{-}028}$ mutant embryos the *schizo* mRNA is still detectable at stage 11 (B) and stage 14 (E) in both tissues (arrow and arrowhead). (C) In embryos homozygous for the *schizo* deficiency Df(3L) *ME178* that lack zygotic *schizo*, the maternal *schizo* transcript is still detectable at stage 11 in the somatic mesoderm when the fusion of myoblasts begins (arrow) and in the central nervous system (arrowhead). (F) At stage 14 the *schizo* mRNA is still present in the central nervous system (arrowhead), but transcription is reduced in the somatic mesoderm (arrow). These data show that the maternal *schizo* mRNA is transcribed when myoblasts begin to fuse.

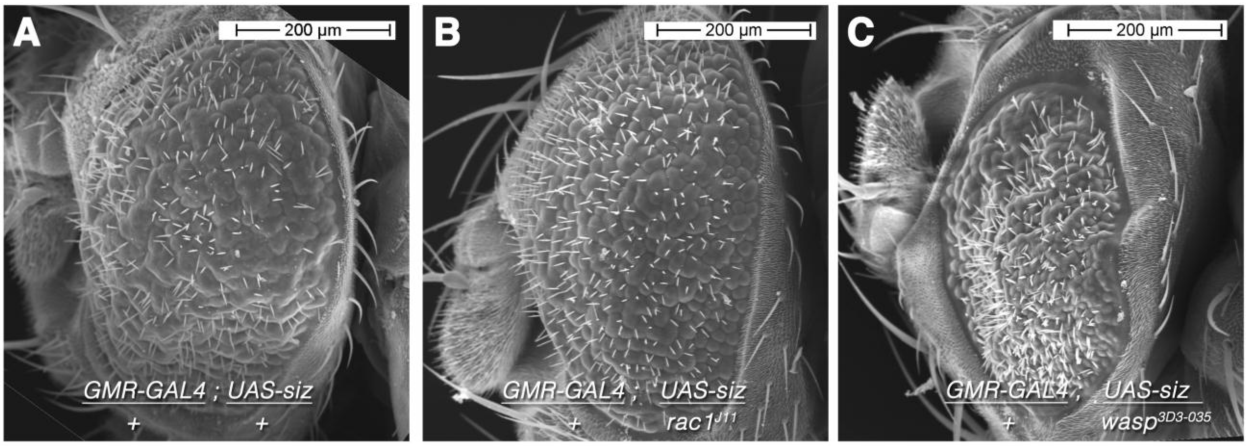

Fig. S7. The loss of *rac* and *wasp* does not suppress the *GMR*-GAL4>>UAS-*siz* induced rough-eye phenotype. Scanning electron micrographs of adult eyes. (A) Rough eye phenotype of heterozygous flies expressing UAS-*siz* under the control of *GMR*-GAL4. (B) Adult eye of a fly expressing heterozygous UAS-*siz* under the control of *GMR*-GAL4 with a reduced *rac* gene dosage. No suppression of the *GMR*-GAL4>>UAS-*siz* induced rough-eye phenotype is observed. (C) Enhancement of the rough-eye phenotype of a fly expressing heterozygous UAS-*siz* under the control of *GMR*-GAL4 with a reduced *wasp* gene dosage.

Biology Open • Supplementary information

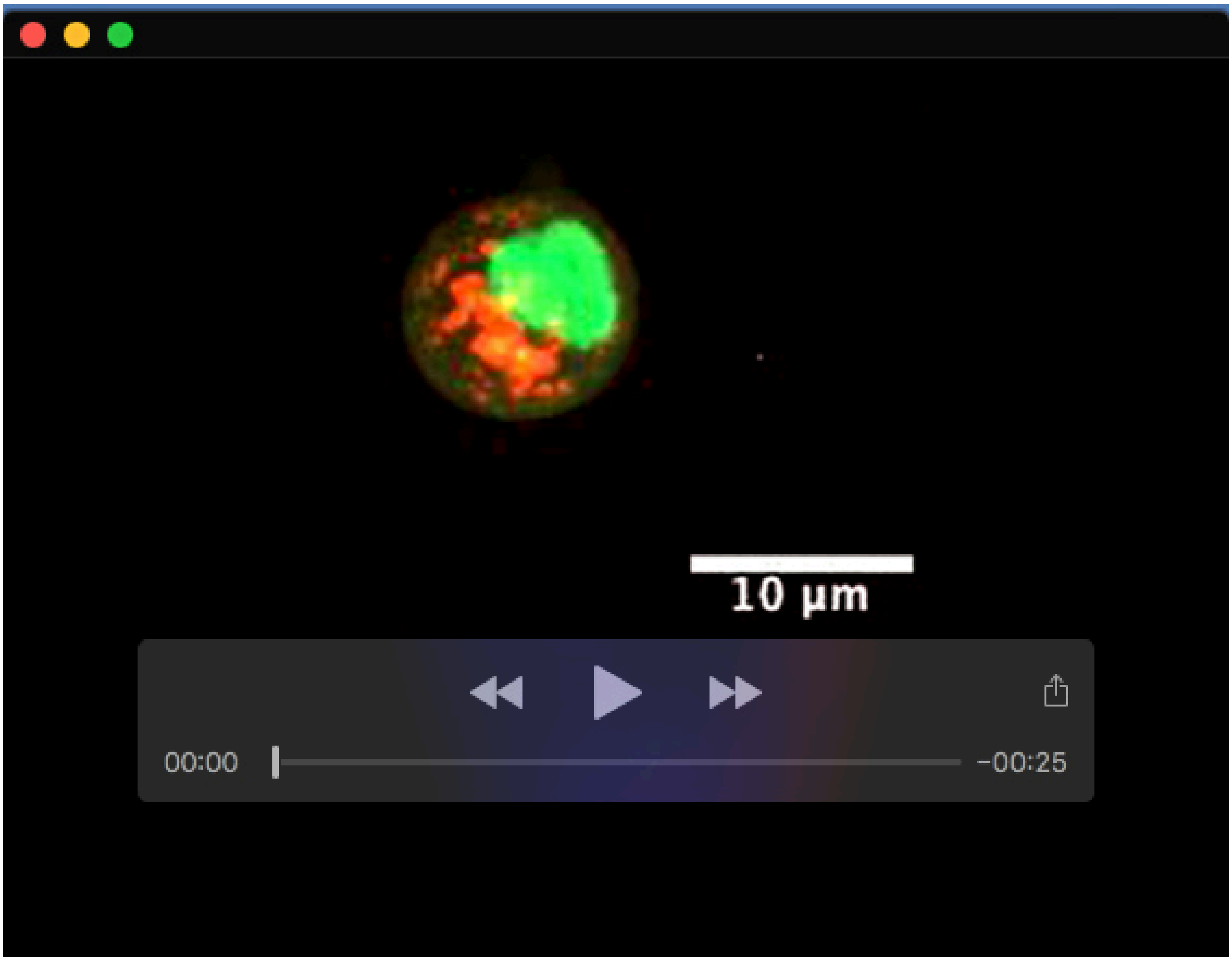

Movie 1.

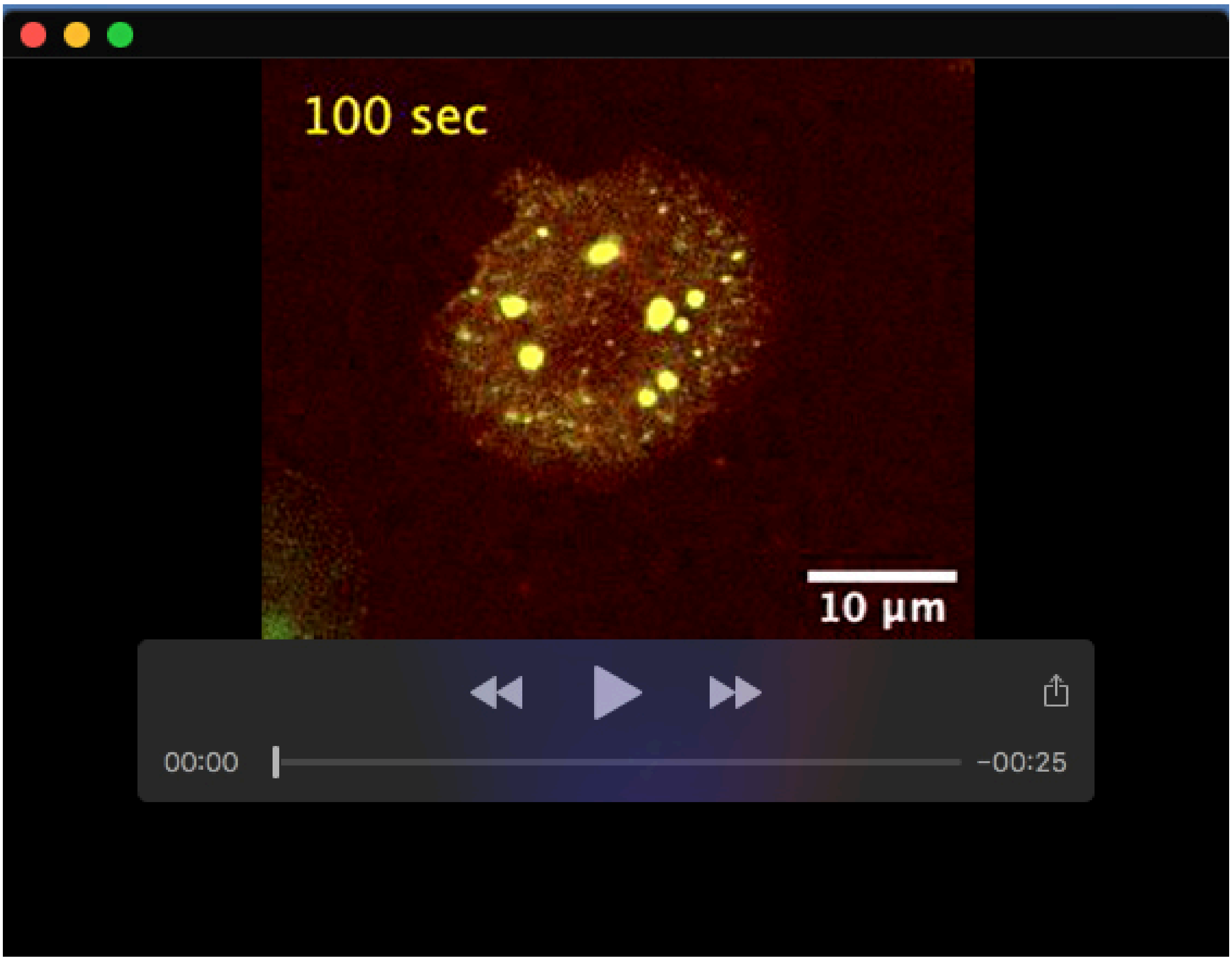

Movie 2.

Biology Open • Supplementary information
